# A Specific Mini‐Intrabody Mediates Lysosome Degradation of Mutant Huntingtin

**DOI:** 10.1002/advs.202301120

**Published:** 2023-09-08

**Authors:** Caijuan Li, Yingqi Lin, Yizhi Chen, Xichen Song, Xiao Zheng, Jiawei Li, Jun He, Xiusheng Chen, Chunhui Huang, Wei Wang, Jianhao Wu, Jiaxi Wu, Jiale Gao, Zhuchi Tu, Xiao‐Jiang Li, Sen Yan, Shihua Li

**Affiliations:** ^1^ Guangdong Key Laboratory of Non‐human Primate Research Key Laboratory of CNS Regeneration (Ministry of Education) GHM Institute of CNS Regeneration Jinan University Guangzhou 510632 China; ^2^ Department of Pathophysiology, School of Medicine Jinan University Guangzhou 510632 China; ^3^ Institute of Laboratory Animal Science Jinan University Guangzhou 510632 China

**Keywords:** HD KI‐140Q mice, intravenous injection, lysosome, mini‐intrabody, mutant huntingtin, SM3, stereotaxic injection

## Abstract

Accumulation of misfolded proteins leads to many neurodegenerative diseases that can be treated by lowering or removing mutant proteins. Huntington's disease (HD) is characterized by the intracellular accumulation of mutant huntingtin (mHTT) that can be soluble and aggregated in the central nervous system and causes neuronal damage and death. Here, an intracellular antibody (intrabody) fragment is generated that can specifically bind mHTT and link to the lysosome for degradation. It is found that delivery of this peptide by either brain injection or intravenous administration can efficiently clear the soluble and aggregated mHTT by activating the lysosomal degradation pathway, resulting in amelioration of gliosis and dyskinesia in HD knock‐in (KI‐140Q) mice. These findings suggest that the small intrabody peptide linked to lysosomes can effectively lower mutant proteins and provide a new approach for treating neurodegenerative diseases that are caused by the accumulation of mutant proteins.

## Graphical abstract

The authors designed a peptide that specifically binds mutant huntingtin and its aggregates, and brings them into lysosomes for degradation, thereby improving neuropathology and behavioral abnormalities in HD KI‐140Q mice.

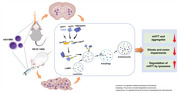

## 1 Introduction

Huntington's disease (HD) is an autosomal‐dominant neurodegenerative disease caused by a single gene mutation in the Huntingtin gene (*HTT*) on chromosome 4.^[^ ^1^ ^]^ The normal *HTT* has less than 36 CAG repeats and encodes a polyglutamine (polyQ) repeat shorter than 36Q in huntingtin protein (HTT), but more than 36Q can lead to abnormal conformation and misfolding of the mutant HTT (mHTT).^[^ ^2^ ^]^ Mutant HTT accumulates in the central nervous system in an age‐dependent manner and causes neuronal damage and death, leading to the development of HD.^[^ ^3^ ^]^ In the brains of HD patients, significant neuronal loss was found in the striatum, with extreme brain atrophy in the middle and late stages of the disease,^[^ ^4^ ^]^ a large number of mHTT aggregates accompanied by dystrophic neurites, which reflects the accumulation of misfolded mHTT that can affect intracellular transport, gene transcription, and neuronal survival.^[^ ^4^ ^]^ Despite considerable advances in our understanding of the pathogenesis of HD, there are currently no effective treatments for HD.

Lowering the levels of misfolded proteins in neurodegenerative diseases is an emerging approach for treatment and can be achieved by using various types of technology such as RNAi‐based microRNA, siRNA, shRNA, and CRISPR/Cas9‐mediated gene inactivation.^[^ ^5^ ^]^ In addition, some researchers also designed an amino acid‐peptide adapter molecule QBP1‐HSC70bm, which uses chaperone‐mediated autophagy to selectively degrade mHTT. It can reduce PolyQ aggregation in striatum of R6/2 mice, improve symptoms, and extend lifespan.^[^ ^6^ ^]^ Recently, a research group designed an amphiphilic peptide (8R10Q) that assembles into nanovesicles and targets mutant huntingtin (mHtt). 8R10Q can disrupt mHtt oligomer assembly process and reduce mHtt toxicity.^[^ ^7^ ^]^

These treatments have been tried to treat HD and provide promising therapeutic effects.^[^ ^8^ ^]^ These treatments basically target mRNA or DNA, and their potential off‐targets are still a considerable concern. In addition, when mutant proteins have already accumulated in the brains, it would be more efficient to target mutant proteins than the disease gene to alleviate neuropathological toxicity of mutant proteins, which may explain, at least partly, for recent failures of clinical trials of ASO to treat HD.^[^ ^9^ ^]^ In this regard, it would also be important to find a new therapeutic method that can efficiently clear or remove mutant protein that has already accumulated.

Intracellular antibody (intrabody) refers to an engineered antibody that is expressed in non‐lymphocytes and can be distributed in subcellular compartments, such as the nucleus and cytoplasm, to target a specific protein.^[^ ^10^ ^]^ Intrabody mainly exists in two forms, scFv (single chain fragment variable) and Fab.^[^ ^10a^ ^]^ Because scFv antibody has a simple molecular structure and maintains the affinity of the antibody to the antigen, it has become the most used form of intrabody.^[^ ^11^ ^]^ In addition, modifications made to the N‐ or C‐terminus of the scFv protein can lead the scFv to be localized and expressed in a specific subcellular compartment.^[^ ^12^ ^]^

Previous studies have reported intrabodies targeting different regions of mHTT, including soluble and aggregated mHTT.^[^ ^13^ ^]^ The targeting regions include polyQ, polyP, and the N‐terminus 17 amino acids in HTT exon1. These intrabodies were found to be able to block soluble or insoluble mHTT.^[^ ^13a^ ^‐^ ^13^, ^14^ ^]^ However, the above‐mentioned intrabodies are all ≈20–30 kDa, making them unable to cross the blood brain barrier, and their efficiency to remove mHTT inside neuronal cells needs to be improved.

In our previous study, we found that an intrabody can be expressed in neurons and reduce the cytotoxicity of N‐terminal mutant HTT in HD mice via intra‐brain injection.^[^ ^14c^ ^]^ We recently identified that the last 23 amino acids of the C‐terminus of the heavy chain of our published intrabody are able to bind mHTT and we named it smaller intrabody 3 (SM3). Since lysosomes are more efficient than the proteasome in degrading large proteins or aggregates, we fused the signal sequence of lysosomal associated membrane protein 1 (LAMP1) to the C‐terminus of SM3, which enables the bound mHTT to be carried to and degraded in the lysosomes.^[^ ^15^ ^]^ To be able to recognize this peptide, we also in frame fused an HA epitope in the N‐terminus of SM3. We further demonstrated that SM3 selectively binds to mHTT and promotes the degradation both of soluble and insoluble mHTT and alleviates the abnormal behavior of HD KI‐140Q mice by increasing the lysosomal degradation activity. These findings suggest that the SM3 has a great potential for treating Huntington's disease.

## 2 Results

### 2.1 SM3 Efficiently Reduces mHTT

**Figure 1:**
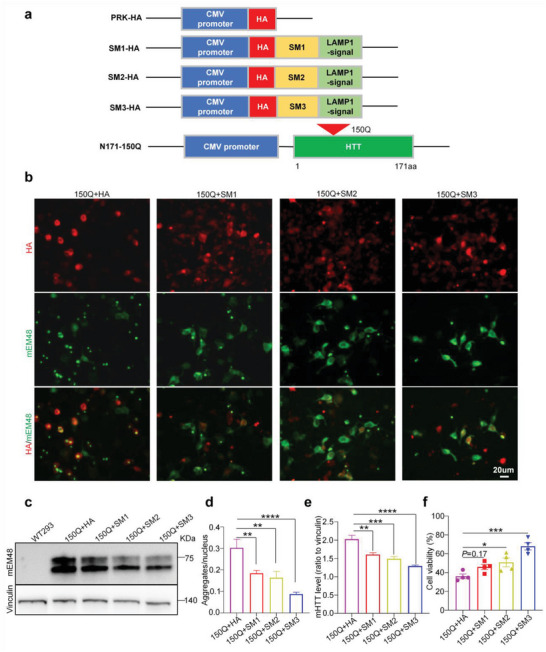
Effects of different intrabody fragments on mHTT. a) Schematics of the intrabody fragments SM1, SM2, and SM3 (24, 29, and 23 amino acids), and N‐terminal mutant HTT fragment (HTT‐N171‐150Q). The intrabody peptides are overlapping fragments of variable region heavy (VH) chain of the scFv‐mEM48, which is tagged with the HA epitope and linked to a LAMP1 signal. N‐terminal mHTT fragment (HTT1–171aa) contains 150 glutamine repeats (150Q). b) Transfection of SM1, SM2, or SM3 with N171‐150Q in HEK 293T cells showed that SM3 can reduce aggregates more efficiently. Scale bar: 20 µm. c) Western blotting analysis of SM1 or SM3 and mutant HTT transfected HEK293T cells using mEM48 antibody. d) Quantification of the aggregates in co‐transfected HEK 293T cells (*n* = 8 images per group). Data were analyzed by one‐way ANOVA with Dunnett's multiple comparisons test and are presented as mean ± SEM. **P = 0.0085 (150Q+HA vs 150Q+SM1); **P = 0.0020(150Q+HA vs 150Q+SM2); ****P < 0.0001 (150Q+HA vs 150Q+SM3). e) Quantification of the ratios of mHTT to vinculin on the western blots. The data were obtained from four independent experiments (*n* = 4). Data were analyzed by one‐way ANOVA with Dunnett's multiple comparisons test and presented as mean ± SEM. **P = 0.0025; ***P = 0.0003; ****P < 0.0001. f) Quantification of HEK293 cell viability using cell counting kit‐8 (CCK8). The data were obtained from four independent experiments (*n* = 4). Data were analyzed by one‐way ANOVA with Dunnett's multiple comparisons test and presented as mean ± SEM. *P = 0.031; ***P = 0.0001.

Since our generated monoclonal mouse HTT antibody (mEM48) can preferentially recognize the mHTT protein, we previously constructed an intrabody that can target mHTT based on the sequences of mEM48.^[^ ^14c^ ^]^ By co‐transfection of the light chain (LC) or heavy chain (HC) of scFv with HTT N‐171‐150Q in 293T cells to determine the binding sites of the intrabody, we found that it was the heavy chain that binds to mHTT. In order to find the region of this intrabody that can specifically bind to mHTT, we designed three overlapping peptides from the heavy chain of mEM48 scFv (70 amino acids), which included peptide 1: amino acids 1–24, peptide 2: amino acids 21–50, and peptide 3: amino acids 47–70. The C‐terminus of these peptides was linked to the lysosomal signaling peptide (GYQTI) for targeting mHTT to the lysosomes for degradation^[^ ^16^ ^]^ (**Figure** **1**a). We named these three peptides as small fragment 1–3, or SM1, SM2, and SM3, respectively. To examine the ability of SM1, SM2, and SM3 to degrade mHTT, we co‐transfected them with N‐terminal mHTT fragments (N171‐150Q) in HEK 293T cells and then examined HTT expression levels via immunofluorescence (Figure 1b,d) and western blotting (Figure 1c,e) analyses. The results showed that SM1, SM2, and SM3 were all able to reduce mHTT and its aggregates with SM3 being the most effective at reducing mHTT. At the same time, in order to detect the impact of intrabody on cell survival, we tested cell viability, and the results showed that SM3 had a certain improvement effect on cell viability (Figure 1f). Therefore, we chose SM3 for subsequent studies. To test if SM3 specifically binds and degrades mHTT, we also co‐transfected SM3 with HTT‐N171‐23Q. The results showed that SM3 did not alter the protein level of HTT‐N171‐23Q but significantly reduced HTT‐N171‐150Q (Figure S1a,b, Supporting Information). This result gives us confidence that SM3 can specifically reduce mHTT and we therefore used it for further studies.

### 2.2 SM3 Mediates mHTT Reduction via Lysosomal Autophagy Activation

**Figure 2:**
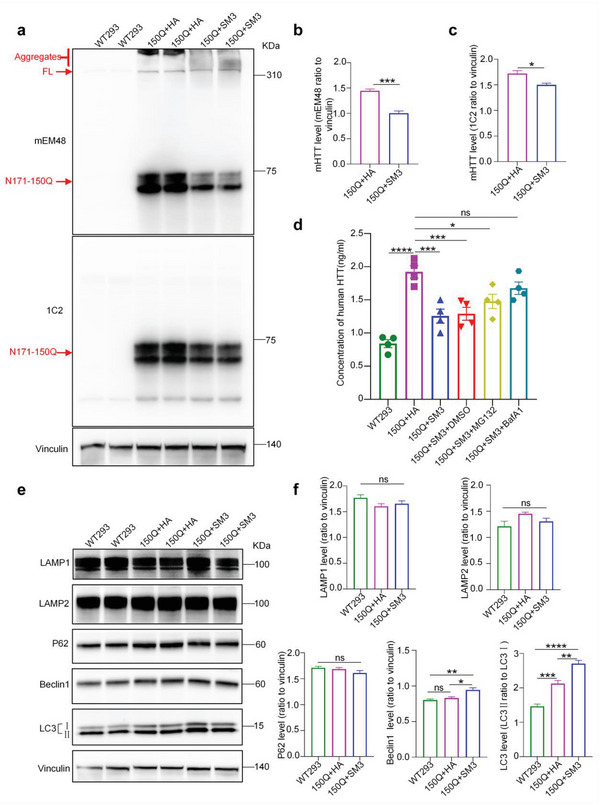
Western blotting of SM3 and HTT‐N171‐150Q transfected 293 cells. a) Western blotting analysis of 293T cells transfected with HTT‐N171‐150Q or HTT‐171‐150Q cot‐transfected with SM3. b,c) Quantification of the ratios of mHTT recognized by mEM48 or 1C2 to vinculin on the western blots. The data were obtained from four independent experiments (*n* = 4). Data were analyzed by two‐tailed Student's *t* test and presented as mean ± SEM. *P = 0.0143; ***P = 0.0002. d) Quantification of human HTT using ELISA. *n* = 4 per group. Data were analyzed by one‐way ANOVA with Dunnett's multiple comparisons test and presented as mean ± SEM. *P = 0.0155; ***P = 0.0005(150Q+HA vs 150Q+SM3); ***P = 0.0008(150Q+HA vs 150Q+SM3+DMSO); ****P <0.0001; ns = 0.2693. e) Western blotting analysis of the transfected 293T cells, probed with LAMP1, LAMP2, P62, Beclin 1, and LC3 antibodies to detect whether the lysosomes‐autophagy pathway was activated. Vinculin served as a loading control. f) Quantification of the ratios of LAMP1, LAMP2, P62, and Beclin 1 to vinculin or LC3II to LC3I on the western blots. The data were obtained from four independent experiments (*n* = 4). Data are analyzed by one‐way ANOVA with Dunnett's multiple comparisons test and presented as mean ± SEM. Beclin 1: *P = 0.0113; **P = 0.0029. LC3: **P = 0.0018; ***P = 0.0008; ****P <0.0001. WT293 = Untransfected 293 cells.

Previous studies of protein degradation have reported that lysosome‐autophagy is the main protein degradation machinery in the cell.^[^ ^17^ ^]^ Because SM3 is linked to the lysosome signal peptide, we wanted to investigate whether the binding of SM3 to mHTT could activate the lysosomal pathway in cells to remove mHTT. Further examination of the SM3‐mediated mHTT degradation by western blotting showed that after transfection of SM3, both the soluble and aggregated HTT‐N171‐150Q were reduced to a certain extent (**Figure** **2**a–c). Moreover, we performed enzyme‐linked immunosorbent assay (ELISA) experiments to detect the degradation of mHTT mediated by SM3. The results indicated that upon transfection with SM3, the level of human HTT was reduced to a significant extent (Figure 2d). We therefore examined the proteins related to the lysosomal function and found that the expression of LC3 was increased to some extent after transfection with SM3 (Figure 2e,f).

Since transient transfection may be unstable, we also tested the ability of SM3 to degrade mHTT in 120Q‐293 cell line that stably expresses full‐length mHTT with 120Q. Western blotting results showed that the full‐length mHTT was significantly decreased after transfection with SM3 (Figure S2a–d, Supporting Information). ELISA experiment results also indicated that the level of human HTT was reduced (Figure S2e, Supporting Information). Further, the lysosomal marker proteins such as LAMP1, LAPM2, P62, Beclin 1, and LC3 were significantly increased after SM3 was expressed in the 293‐HTT‐120Q stable cell line (Figure S2f,g, Supporting Information). This finding suggests that SM3 expression may have induced lysosome‐autophagy activation to promote the degradation of mHTT.

### 2.3 SM3 Brings mHTT into the Lysosome for Degradation

**Figure 3:**
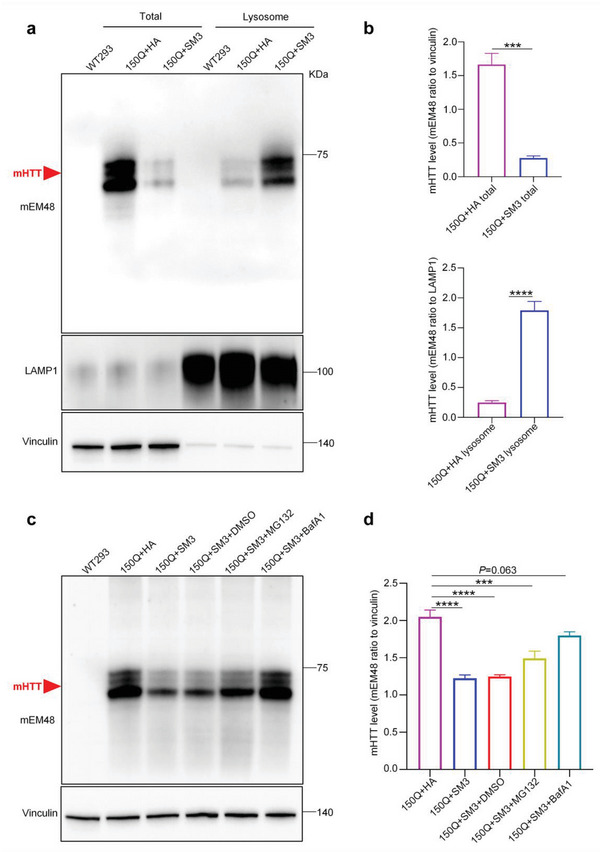
Lysosomal fractionation of SM3 and mHTT‐transfected 293T cells. a) Western blotting analysis of transfected mHTT and SM3 with mEM48 antibody to detect HTT‐N171‐150Q. The blots were also probed with LAMP1 and Vinculin antibodies. b) Quantification of the ratios of mHTT to vinculin (total protein) or LAMP1 (lysosome) on the western blots. The data were obtained from four independent experiments (*n* = 4). Data were analyzed by two‐tailed Student's *t* test and presented as mean ± SEM. ***P = 0.0002; ****P <0.0001. c) Western blotting analysis of mHTT, which in transfected 293T cells with SM3 and added proteasome inhibitor MG132 or lysosome inhibitor bafA1. HA transfection was used as control for SM3. d) Quantification of the ratios of mHTT to vinculin on the western blots. The data were obtained from four independent experiments (*n* = 4). Data were analyzed by one‐way ANOVA with Dunnett's multiple comparisons test and presented as mean ± SEM. ***P = 0.0001; ****P <0.0001. WT293 = un‐transfected 293 cells.

To further verify whether the SM3 can bring mHTT to lysosomes for degradation, we purified lysosome fraction from 293T cells transfected with mHTT or mHTT and SM3. We found that cells transfected with mHTT plus SM3 contained a large amount of mHTT in the purified lysosome fraction, compared to the cells transfected with mHTT and a control vector (HA only in the vector). Consistently, the intracellular mHTT was significantly reduced after transfection with SM3 (**Figure** **3**a,b). We also verified the purity of the lysosome fraction with the lysosomal marker proteins (Figure 3a). In addition, we used western blotting to examine (Figure 3c,d) and ELISA (Figure 2d) experiments that the efficiency of SM3 in degrading mHTT was significantly reduced in the presence of the lysosomal inhibitor bafilomycin A1 (bafA1), while the degradation efficiency of SM3 was only slightly affected in the presence of the proteasome inhibitor MG132. These results demonstrate that SM3 can specifically bring mHTT protein into the lysosome and activate the lysosome‐autophagy pathway to promote mHTT degradation.

### 2.4 SM3 Reduces Mutant HTT Expression in the Brains of HD KI‐140Q Mice

**Figure 4:**
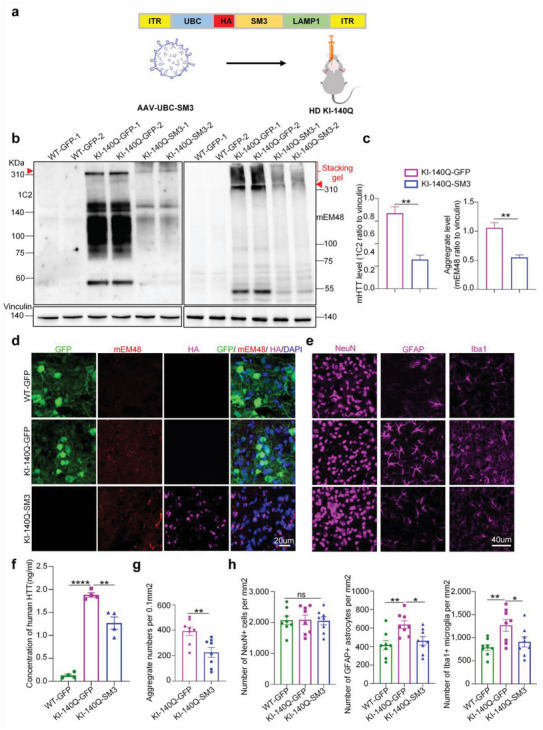
Analysis of HD KI‐140Q mice after stereotaxic injection with SM3. a) Schematic diagram of AAV‐PHP.eB‐HA‐SM3 viral vector for the brain injection, which expresses HA‐tagged SM3 under the control of the UBC promoter. b) Western blotting analysis of the striatum of HD KI‐140Q and wild type (WT) mice one month after stereotaxic injection with GFP (control) or SM3. Western blots were probed with 1C2 or mEM48 antibody to detect mHTT and its aggregates. Representative western blots show samples from two animals in each group. mHTT aggregates are in the stacking gel, the full‐length mutant HTT is indicated by an arrowhead. c) Quantification of the ratios of mHTT to vinculin on the western blots. The data were obtained from four independent experiments (*n* = 4). Data were analyzed by two‐tailed Student's *t* test and are presented as mean ± SEM. **P = 0.001 (1C2); **P = 0.002. d) Immunofluorescent images of brain sections stained with antibodies mEM48, GFP, and HA (SM3). DAPI is used for nuclear staining. Scale bars: 20 µm. e) Representative immunofluorescent fluorescent images of the striatum from GFP or SM3 injected HD KI‐140Q mice. Antibodies for NeuN, GFAP, and Iba1 were used. Scale bars: 40 µm. f) ELISA assay of human HTT. *n* = 4 mice per group. Data were analyzed by one‐way ANOVA with Dunnett's multiple comparisons test and presented as mean ± SEM. **P = 0.0011; ****P < 0.0001. g) Quantification of mHTT aggregates in SM3‐injected mice. Cells expressing mHTT aggregates were counted per 0.1mm^2^, *n* = 8 mice per group. Data are analyzed by two‐tailed Student's *t* test and presented as mean ± SEM. **P = 0.0056. h) Quantification of the NeuN, GFAP, Iba1 immunostaining. Data are analyzed by one‐way ANOVA with Dunnett's multiple comparisons test and presented as mean ± SEM. *n* =  8 mice per group; GFAP: *P = 0.0262 (WT GFP verses HD KI‐140Q+SM3); **P = 0.0056 (WT GFP verses HD KI‐140Q). Iba1: *P = 0.0448 (WT GFP verses HD KI‐140Q +SM3); **P = 0.0071 (WT GFP verses HD KI‐140Q). Note that the numbers of NeuN‐positive cells were not changed in all scenarios but injection of SM3 decreased the GFAP or Iba1 positive cells.

Although the above results demonstrated that the SM3 can effectively reduce intracellular mHTT, it remains to be investigated whether it can reduce mHTT protein in the HD KI‐140Q mouse brain. In HD KI‐140Q mice, mHTT is first accumulated in the striatum of the brain and forms noticeable aggregates at 4 months of age.^[^ ^18^ ^]^ To express SM3 in HD mouse brains, we packaged AAV‐PHP.eB virus expressing HA‐SM3 and stereotaxically injected it into the striatal parenchyma of 6‐month‐old HD mice bilaterally (**Figure** **4**a), AAV‐PHP.eB‐GFP was injected as a control. One month after the injection, mice were euthanized to collect brain tissues for analysis. Western blotting results showed that both soluble and aggregated mHTT (Figure 4b,c) were significantly reduced after SM3 injection. The immunofluorescence staining of HA‐tagged‐SM3 showed that SM3 was efficiently expressed in the mouse brain (Figure 4d). Also, mEM48 immunofluorescence staining results showed that the level of aggregated mHTT in the brains of HD mice was significantly reduced after injection of SM3 (Figure 4d,g). Moreover, the ELISA results indicated that upon injection of SM3, the level of human HTT was reduced to a significant extent (Figure 4f).

In HD KI‐140Q mice, mHTT accumulation is accompanied by reactive gliosis.^[^ ^19^ ^]^ Therefore, we examined whether increased gliosis was improved after SM3 injection. Compared to the control injection, immunofluorescence staining and western blotting results showed that injection of SM3 in the striatum of HD KI mice significantly decreased the numbers of reactive astrocytes and microglia but did not change the number of NeuN positive staining cells (Figure 4e,h; Figure S3a,b, Supporting Information). These results indicate the numbers of astrocytes and microglia have been reduced by injecting the SM3. The similar effects to reduce gliosis by SM3 injection were also verified in HD KI mice at 6 months of age (Figure S3a,b, Supporting Information). Thus, these results indicate that reactive gliosis in HD KI‐140Q mice was effectively alleviated by SM3 treatment.

### 2.5 SM3 Activates Lysosomes and Amelioration of Dyskinesia in HD KI‐140Q Mice by Stereotaxic Injection

**Figure 5:**
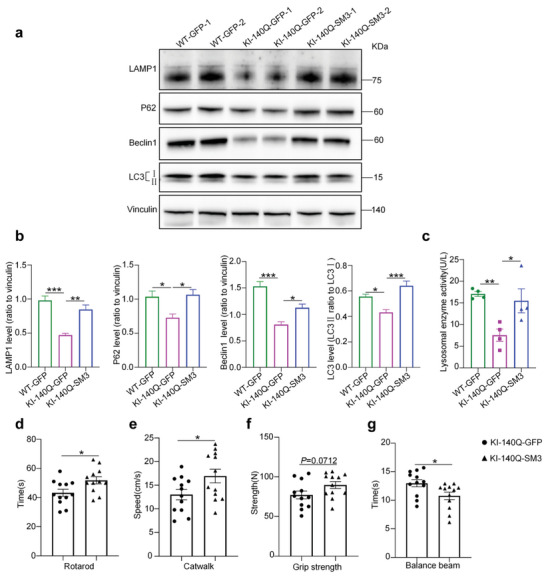
Western blotting and behavior examination of HD KI‐140Q mice injected with SM3. a) Western blotting analysis of the striatum of HD KI‐140Q, injected with GFP (Control) or SM3, and wild type (WT) mice injected with GFP at 7 months of age. Antibodies to LAMP1, P62, Beclin 1, and LC3 were used. Vinculin served as a loading control. b) Quantification of the ratios of LAMP1, P62, and Beclin 1 to vinculin or LC3II to LC3I on the western blots. The data were obtained from four independent experiments (*n* = 4). Data were analyzed by one‐way ANOVA with Dunnett's multiple comparisons test and presented as mean ± SEM. LAMP1: ***P = 0.0003; **P = 0.0021. P62: *P = 0.0266 (WT‐GFP vs KI‐140Q‐GFP); *P = 0.0169 (KI‐140Q‐GFP vs KI‐140Q‐SM3). Beclin 1: *P = 0.0215; ***P = 0.0001. LC3: *P = 0.0203; ***P = 0.0009. c) Quantification of lysosomal enzyme activity using ELISA. *n* = 4 mice per group. Data were analyzed by one‐way ANOVA with Dunnett's multiple comparisons test and presented as mean ± SEM. **P = 0.0093; *P = 0.0241. d–g) Motor functions of HD KI‐140Q mice injected with GFP (KI‐140Q‐GFP) as control or with SM3 (KI‐140Q‐SM3). Mice were injected at 6 months of age and examined one month later. Mice were examined by rotarod, catwalk, grip strength, and balance beam tests one month after brain injection (*n* = 12 mice per group). Data were analyzed with two‐tailed Student's *t* test and presented as mean ± SEM. *P = 0.0211 (rotarod); *P = 0.0405 (catwalk); *P = 0.0232 (balance beam).

To test whether the reduced mHTT level in HD mouse brain was due to mHTT degradation by lysosomal‐autophagy activation and since SM3 can bring mHTT to the lysosomes, we examined if there was any alteration of the lysosomal‐autophagy proteins, including LAMP1, P62, Beclin 1 and LC3. We found that these proteins were decreased in HD KI‐140Q mice and that SM3 injection could reverse this decrease (**Figure** **5**a,b), which suggests that the lysosomal function may be impaired in HD mice but SM3 could activate the lysosome function because of its targeting to the lysosomes. To confirm this, we examined the lysosome activity in the brain tissues using ELISA. The results showed that injection of SM3, indeed improved the acid enzymatic activity of the lysosome in HD KI‐140Q mouse brain tissues to the level similar to that of WT mouse lysosomes (Figure 5c).

Previous studies indicated that HD KI‐140Q mice displayed impairment of exercise capacity and reduced muscle strength.^[^ ^18^, ^20^ ^]^ Given that SM3 significantly reduced mHTT‐related pathology, we assessed its beneficial effects on the motor functions, including endurance exercise, exercise speed, grip strength, and motor balance, of HD KI‐140Q mice using the rotarod test, the catwalk test, the grip strength test, and the balance beam test, respectively.

In the rotarod experiment, HD KI‐140Q mice treated with SM3 ran longer on a rotarod and exhibited better exercise tolerance than the GFP‐injected (control) HD KI‐140Q mice (Figure 5d). The catwalk experiment measures the average speed of mice through a specific distance. Compared with GFP‐injected mice, HD KI‐140Q mice injected with SM3 showed a significant increase in the average speed over a certain distance (Figure 5e). In the grip strength experiment that evaluated the muscle strength, the muscle strength of HD KI‐140Q mice injected with SM3 was improved to a certain extent compared with HD mice injected with GFP (Figure 5f). In the balance beam experiment, HD KI‐140Q mice injected with SM3 took a shorter time to walk across the beam (Figure 5g). In summary, our results indicated that the brain injection of SM3 can significantly improve the motor ability of HD KI‐140Q mice.

### 2.6 Intravenous Injection of SM3 Reduced mHTT and Neuropathology in HD KI‐140Q Mice

**Figure 6:**
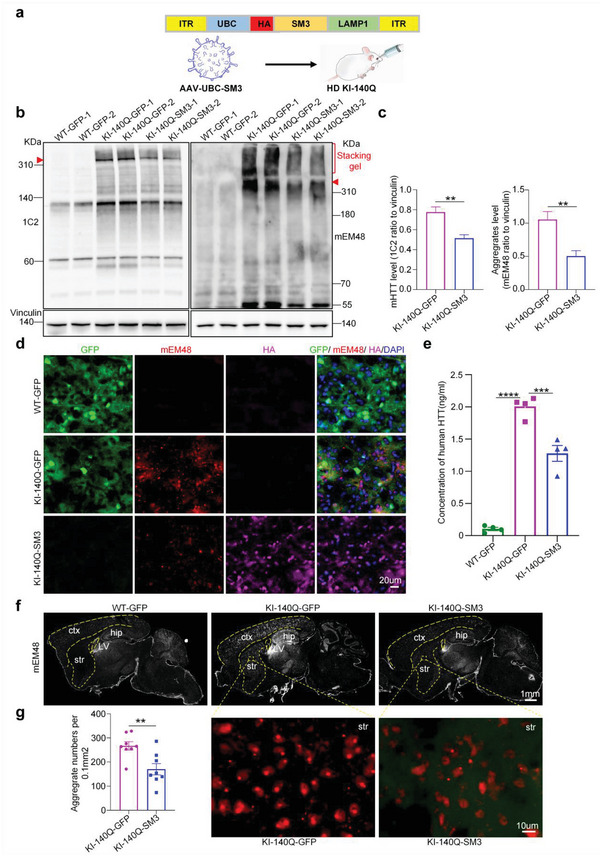
Analysis of the brains of HD KI‐140Q mice after intravenous injection of SM3. a) Diagram of GFP or SM3 virus for intravenous injection. b) Western blotting analysis of mHTT in the HD KI‐140Q and WT mice injected with GFP or SM3 one month after injection, 1C2 antibody was used to detect full‐length mHTT, and mEM48 antibody to detect aggregates (stacking gel) and full‐length mHTT (arrowhead). c) Quantification of the ratios of mHTT to vinculin on the western blots. The data were obtained from four independent experiments (*n* = 4). Data were analyzed by two‐tailed Student's *t* test and presented as mean ± SEM. **P = 0.0059 (1C2); **P = 0.0089 (mEM48). d) Immunofluorescent staining using antibodies to GFP, mHTT (mEM48), and HA (HA‐SM3) in the striatum of HD KI mice one month after the injection. Scale bars: 20 µm. e) ELISA assay of human HTT. *n* = 4 mice per group. Data were analyzed by one‐way ANOVA with Dunnett's multiple comparisons test and presented as mean ± SEM. ***P = 0.0004; ****P < 0.0001. f) Representative whole brain sagittal images of mEM48 staining in the HD KI‐140Q mice injected with GFP or SM3. ctx: cortex; str: striatum; hip: hippocampus; LV: lateral ventricle. Scale bars: 1 mm. g) Representative images of aggregate (mEM48) staining in the HD KI‐140Q mouse striatum and quantification of aggregates. Scale bars: 10 µm. Data were analyzed by two‐tailed Student's t test and presented as mean ± SEM. *n* =  8 mice per group; **P =  0.005.

Intravenous injection of intrabody will be more acceptable and convenient for clinical treatment. We sought to use ophthalmic vein injection of SM3 to test whether this method can also result in SM3 expression in the animal brain. According to previous reports, AAV‐PHP.eB can efficiently cross the blood‐brain barrier.^[^ ^21^ ^]^ We packaged SM3 with the PHP.eB serotype package vector and delivered the AAV(PHP.eB)‐GFP or SM3 to HD KI‐140Q mice via superior ophthalmic vein injection (**Figure** **6**a). Mice were euthanized one month later, and their brains were collected for experimental analysis. SM3 was widely distribute in the whole brain of 6‐month‐old HD KI‐140Q mice (Figure S4, Supporting Information). Compared with the control HD KI‐140Q mice injected with GFP, HD KI‐140Q mice injected with SM3 showed that full‐length mHTT protein and aggregated mHTT were significantly reduced (Figure 6b,c). Immunofluorescence staining of the mouse brain showed that the striatum of mice injected with expressed the HA‐tagged SM3 (Figure 6d; Figure S4, Supporting Information). The ELISA results showed that the level of human HTT was reduced to a significant extent after vein injection of SM3 (Figure 6e). Consistently, immunofluorescent staining with anti‐HTT (mEM48) showed that the aggregates produced by mHTT were significantly reduced in the whole brain, especially in the striatum (Figure 6f,g). Thus, intravenous delivery of SM3 was also able to effectively reduce mHTT in the brain.

We then explored whether intravenous delivery of SM3 into the brain could reduce neuropathology in HD KI‐140Q mice. Although the NeuN protein level in the striatum of HD KI‐140Q mice was not changed by intravenous administration of SM3 (Figure S5d,e, Supporting Information), the intensity of GFAP (Figure S5d,e, Supporting Information) and Iba1 (Figure S5d,e, Supporting Information) labeling on western blots was significantly reduced in HD mice after intravenous injection of SM3. Immunofluorescent staining experiments also showed similar alleviation effect of reactive gliosis by SM3 (Figure S5b,c,f, Supporting Information). These results suggest that reactive gliosis in HD KI‐140Q mice was significantly reduced after intravenous injection of SM3.

### 2.7 Intravenous Injection of SM3 Promoted mHTT Degradation and Alleviated HD KI Mouse Behavior Deficits

**Figure 7:**
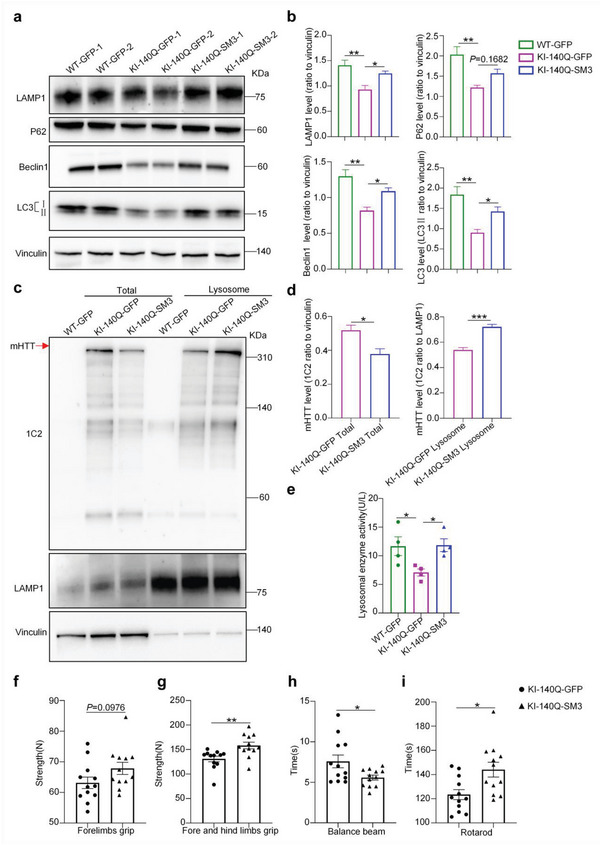
Western blotting and behavior analysis of HD KI‐140Q mice intravenously injected with SM3. a) Western blotting analysis of HD KI‐140Q and WT mice injected with GFP or SM3 using antibodies to LAMP1, P62, Beclin 1, and LC3. Vinculin served as a loading control. b) Quantification of the ratios of LAMP1, P62, Beclin 1, and LC3II to vinculin on the western blots. The data were obtained from four independent experiments (*n* = 4). Data were analyzed by one‐way ANOVA with Dunnett's multiple comparisons test and presented as mean ± SEM. LAMP1: **P =  0.0040; *P =  0.0359. P62: **P =  0.0038. Beclin 1: **P =  0.0011; *P =  0.0302. LC3: **P =  0.0020; *P =  0.0481. c) Western blotting analysis of the HD KI‐140Q mouse striatum to detect mHTT in the purified lysosome fraction. Full‐length mHTT was detected by 1C2 antibody and indicated by an arrow. The blots were also probed with LAMP1 and vinculin antibodies. d) Quantification of the ratios of mHTT to vinculin (total protein) or LAMP1 (lysosome) on the western blots. The data were obtained from four independent experiments (*n* = 4). Data were analyzed by two‐tailed Student's *t* test and presented as mean ± SEM. *P =  0.0174; ***P =  0.0006. e) ELISA assay of lysosomal enzyme activity. *n* = 4 mice per group. Data were analyzed by one‐way ANOVA with Dunnett's multiple comparisons test and presented as mean ± SEM. *P = 0.0424 (WT‐GFP vs KI‐140Q‐GFP); *P = 0.0358 (KI‐140Q‐GFP vs KI‐140Q‐SM3). f–i) Evaluation of motor functions of HD KI‐140Q mice one month after intravenous injection of GFP (KI‐140Q‐GFP) or SM3 (KI‐140Q‐SM3) using f) forelimbs grip, g) fore and hind limbs grip, h) balance beam, and i) rotarod tests. Data were analyzed by two‐tailed Student's *t* test and presented as mean ± SEM. **P = 0.005 (fore and hind limbs grip); *P = 0.0279 (balance beam); *P = 0.0105 (rotarod). *n* = 12 mice per group.

Since brain injection of SM3 resulted in lysosome‐autophagy activation in the HD KI‐140Q mouse brain, we also examined the lysosome‐autophagy related proteins in HD KI‐140Q mice one month after the injection. Similar to the brain‐injected KI‐140Q mice, intravenous SM3‐injected mouse brain tissues also showed the restoration of the LAMP1, P62, Beclin 1, and LC3 protein levels (Figure 7a,b). Western blot results showed that the amount of mHTT in the purified lysosomes from HD KI‐140Q mice injected with SM3 was increased when compared to that in the control HD KI‐140Q mice injected with GFP (**Figure** **7**c,d). ELISA assay demonstrated that the lysosomal enzyme activity was increased in the brain tissues of SM3‐injected HD KI‐140Q mice (Figure 7e), also supporting the idea that intravenous injection of the SM3 can induce lysosomal activation and mHTT degradation in the brain.

We also performed behavior tests such as muscle strength and exercise capacity in mice following intravenous injection of SM3. In the forelimb grip strength test, HD KI‐140Q mice treated with SM3 showed a certain degree of enhancement (Figure 7f). HD KI‐140Q mice also had significantly increased muscle strength as shown on the grip strength test results (Figure 7g). Also, SM3‐treated mice took less time to cross the balance beam (Figure 7h) and stayed longer on the rotarod (Figure 7i). These results indicate that SM3 can be delivered via intravenous injection to the HD KI‐140Q mice to effectively improve the motor functions.

### 2.8 SM3 Mitigated mHTT‐Mediated Gene Transcription Dysregulation

**Figure 8:**
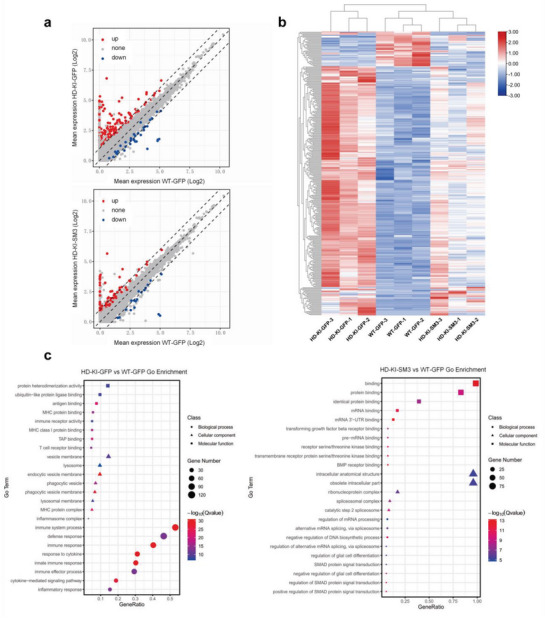
RNA‐seq analysis of HD KI mice injected with SM3 or GFP. a) Scatter plots of differentially expressed genes (DEGs). The striatal mRNA expression of HD‐KI mice treated with SM3 is compared with those of the HD‐KI or WT mice treated with GFP (padj ≤ 0.05; fold change ≥ ± 2.0; *n* = 3). b) Heat map of DEGs expression in the striatum of wild type, HD KI‐140Q, and the SM3‐treated HD KI‐140Q mice. c) GO enrichment analysis of differentially expressed genes in the SM3‐treated and control HD KI‐140Q mice (padj ≤ 0.01).

HD knock‐in mice show gene transcriptional dysregulation that can be related to immune and inflammatory response.^[^ ^22^ ^]^ We wondered whether reducing mHTT expression via SM3 could ameliorate such molecular defects. Because the striatum is the most affected brain region in HD, we analyzed gene expression profiling in the mouse striatum. Thus, the mice of WT injected with GFP, HD KI‐140Q injected with GFP, and HD KI‐140Q injected with SM3 (*n* = 3 per group) were chosen for investigation. Their brain striatal tissues were then isolated for bulk RNA‐seq analysis, which showed differentially expressed genes (DEGs) in these three groups of mice (**Figure** **8**a,b). HD‐KI 140Q mice with GFP had many abnormally overexpressed genes, while SM3 treatment could correct some of those aberrantly overexpressed genes (Figure 8b). GO enrichment analysis showed that the immune and inflammatory related‐pathways are highly enriched in GFP treated HD‐KI mice, but not in SM3 treated HD‐KI mice (Figure 8c).

Those immune and inflammatory responses caused by mHTT were suppressed in the SM3‐treated HD mice (Figure 8c; Figure S6, Supporting Information). Taken together, these results indicated that SM3 treatment could mitigate gene transcription dysregulation resulted from mHTT expression, consistent with the reduction of mHTT by SM3 in the HD mouse brain.

## 3 Discussion

In this study, we created a small intrabody peptide (SM3) that can efficiently reduce mHTT protein by bringing mHTT to the lysosome for degradation. Moreover, both brain stereotaxic injection and intravenous administration of SM3 effectively reduced soluble and insoluble mHTT in the brains of HD KI‐140Q mice and ameliorated HD‐related neuropathology and motor function deficits. Furthermore, delivering SM3 into the brains of HD KI mice can also mitigate gene transcriptional dysregulation that is related to abnormal immune and inflammatory responses. Overall, the SM3 offers a new therapeutic strategy to treat HD.

Previously characterized intrabodies were 150–250 amino acids (20–30 kDa) in size, depending on whether the light chain or heavy chain or both are used (26‐33), making them difficult to cross the blood‐brain barrier and to enter the nucleus. In our study, we narrowed down the binding region of the previously developed intrabody from mEM48, a monoclonal antibody that selectively binds mHTT but not WT HTT.^[^ ^14c^ ^]^ Therefore, we were able to generate SM3 (23 amino acids), a much smaller intrabody fragment (2.7 kDa) that maintains the property to selectively bind mHTT. With the lysosome signal peptide and the HA tag together, the combined peptide is ≈5 kDa. The small size of this peptide would allow for its easy access to the brain with widespread expression in different brain regions. Since wild type HTT is an indispensable scaffolding protein that plays an important role in normal physiological functions,^[^ ^23^ ^]^ the selective binding of SM3 to mHTT is the key to effective and safe treatment of HD, as wild type HTT would not bind the SM3 so that its normal function would not be interfered. Thus, the use of a small peptide derived from the intrabody that can selectively bind mutant proteins is an obvious advantage over the gene targeting approach that may not unambiguously distinguish the normal and mutant alleles.

We used HD KI mice at the age of 6 months to examine whether SM3 can reduce mHTT. When HD KI mice are at the age of 6 months, there is no obvious alteration in NeuN, GFAP, and Iba1. Especially, no obvious loss of NeuN has been reported in the HD mouse brain even at old ages (> 1 year).^[^ ^5^ ^]^ However, the reduction of mutant HTT is key to the treatment of HD. Using HD KI mice, we can test whether intrabody can reduce mutant HTT at the presymptomatic stage, which is critical for future clinical treatment of HD.

From a broader perspective, we have demonstrated the concept of targeting mutant protein (mHTT) for the lysosome‐mediated degradation via SM3, a small peptide derived from intrabody. Early studies have also tried to direct the intrabodies into autophagic/lysosomal pathway to degrade the bound and misfolded proteins.^[^ ^6^, ^24^ ^]^ However, these treatments were delivered by direct brain injection, and whether these intrabodies can be used for systemic administration via intravenous injection remains unknown. Previous studies have developed a similar strategy by identifying small compounds that interact with both the autophagosome protein and mutant HTT for efficient autophagic clearance of polyQ proteins.^[^ ^25^ ^]^ Based on the idea that small molecules can more readily cross the blood brain barrier and distribute widely in the brain, we generated a small peptide from the HTT‐binding region in an intrabody and demonstrated that tagging this small peptide with the lysosomal recognition signal LAMP1 would also allow the effective clearance of mutant HTT in the brain when it was delivered through ophthalmic vein injection. We also found that this targeting could stimulate the activity of the lysosome, a benefit for rapid removal of mutant proteins.

Previous studies have designed Lysosome‐Targeting Chimeras (LYTACs) that can simultaneously bind to the extracellular domain of membrane proteins, or extracellular proteins, as well as the lysosome‐targeting receptors (LTRs) located on the cell surface, inducing the internalization and lysosomal degradation of target proteins and promoting the targeted degradation of extracellular and membrane‐associated proteins.^[^ ^26^ ^]^ This LYTAC strategy has shown great potential for degrading various target proteins. However, in the case of mHTT protein and aggregates, which are mainly located intracellularly, the lysosome‐targeting signal carried by SM3 can specifically recognize Lysosome‐Associated Membrane Protein 1 (LAMP1), allowing it to function intracellularly and achieve targeted lysosomal degradation of intracellular mutant proteins.

Many neurodegenerative diseases such as Alzheimer's disease (AD), Parkinson's disease (PD), and amyotrophic lateral sclerosis (ALS) are caused by protein misfolding, which results in cytotoxic misfolded proteins or insoluble aggregates in the brain. These misfolded proteins cause neuronal cell death in an age‐dependent manner. Although blocking or lowering the expression of mutant genes is widely accepted as an effective gene therapy for treating neurodegenerative diseases, the majority of neurodegenerative diseases are sporadic and only a small fraction of them carried genetic mutations that may be effectively treated with gene therapy. In addition, recent clinical failures of antisense oligonucleotide treatment of HD highlight the need to use alternative strategy for treatment.^[^ ^9^ ^]^ Thus, clearance of misfolded proteins should be an effective means to treat neurodegenerative diseases. An intrabody small peptide that can specifically bind mutant or misfolded proteins would be a valuable tool for achieving the selective removal of mutant proteins. By developing a small intrabody fragment with the lysosomal targeting signal, our findings suggest that intrabodies can be modified to effectively treat neurodegeneration and other diseases that are caused by misfolded proteins.

## 4 Experimental Section

### Study Design

The objective of this study is to design a small intracellular peptide (SM3) to bind mHTT, which can specifically recognize and bind soluble mHTT protein and insoluble aggregates, and bring them into lysosomes for degradation, which in turn improves neuropathological features and motor impairment in Huntington's disease mice.

This study first validated the efficacy of the constructed plasmids with transient expression of mHTT and SM3 in 293T cells and 120Q‐293T cell lines. The subsequent in vivo experiments used HD KI‐140Q mice to explore its therapeutic effects, which resulted in pathological and behavioral improvement. All mouse experiments were approved by the Institutional Animal Care and Use Committee of Jinan University.

### Antibodies used in the Study

The following primary antibodies were used: anti‐HTT (mEM48) antibody (1:50, MAB5374, Millipore); anti‐polyglutamine antibody (1C2) (1:2000, MAB1574, Millipore); anti‐Huntingtin antibody(EPR5526)(1:5000, ab271195, Abcam); anti‐vinculin antibody (1:1000; MAB3574, Millipore); anti‐NeuN antibody (1:1000, ab177487, Abcam); anti‐GFAP antibody (1:5000, ab7260, Abcam); and anti‐Iba1 antibody (1:1000, 019–19741, Wako); anti‐P62 antibody (1:1000, ab56416, Abcam); anti‐LAMP1 antibody (1:1000, ab24170, Abcam); anti‐LAMP2 antibody (1:1000; 49067S, Cell Signaling Technology); anti‐LC3A/B antibody (1:1000; 4108S, Cell Signaling Technology); Anti‐GFP antibody (1:1000, A11122, Invitrogen); anti‐HA tag antibody (1:100; 3724S, Cell Signaling Technology); anti‐Beclin 1 antibody (1:1000; 3738S, Cell Signaling Technology)

### Plasmid Construction

The small intrabody fragments (SM) DNAs encoding SM1 (24 aa), SM2 (29 aa), and SM3 (23 aa) were chemically synthesized by IGE Biotechenology (Guangzhou China) and were cloned in the PRK5 vector with the CMV promoter to drive its expression, and control plasmid only expresses HA. The plasmids express HTT‐N171‐23Q/150Q are also in the PRK vector. Huntingtin N‐terminal 1–171 amino acid sequences, including 23/150 polyglutamine repeats, were cloned by amplification of HTT with respective polyCAG DNA as template with the primers: forward, 5′‐  GGATCCGCCATGGCTACGTTAGAGAAATTAATG‐3′ and reverse, 5′‐ GGATTCTAAT CTTCCAAGGTTACAGCTCTAGGAATTC‐3′. The PCR condition was as following: 96 °C for 3 min to denature the templates, 80 °C for 30 s for the addition of Taq polymerase (Roche, Expand High Fidelity PCR) and the primers, then 96 °C for 45 s, 60 °C for 45 s, 72 °C for 1 min and the process was repeated for 35 times with 7 min final extension. PCR products were gel purified and ligated with the linearized PRK vector with T4 ligase (Invitrogen) at Bam H1 and EcoR1 cleavage sites.

### Lysosomal Fraction Analysis and Western Blot Analysis

Lysosomal fraction was purified with a commercial kit from BestBio (BB‐3603, Shanghai, China) by following the manufacturer's instructions. The harvested cells and mouse brain tissues were resuspended in cold lysosomal extraction buffer A, and the tissue or cells were grinded using a glass Dounce homogenizer (Sigma‐Aldrich). The lysates were centrifuged at 1000 × g, 3000 × g, and 5000 × g each time for 10 min at 4 °C, respectively. The precipitates were discarded after each span. The supernatant from 5000 × g span was centrifuged at 20 000–30 000 × g again at 4 °C for 20 min in a Beckman high speed centrifuge (Beckman‐Coulter Avanti JXN‐30), and the pellet was collected and resuspended in the lysosome extraction buffer B. The suspension was centrifuged one more time at 20 000–30 000 × g for 20 min at 4 °C. After careful removal of the supernatant, the final pellet was used as the lysosome fraction for western blotting. An equal amount of protein from each sample was loaded on to the acrylamide gel for western blotting analysis.

For western blotting, the harvested cells and mouse brain tissues were grinded by Luka Grinding instrument (LUKYM‐II, China) and lysed in ice‐cold RIPA buffer (50 mm Tris, pH 8.0, 150 mm NaCl, 1 mm EDTA pH 8.0, 1 mm EGTA pH 8.0, 0.1% SDS, 0.5% DOC, and 1% Triton X‐100) containing Halt Protease Inhibitor cocktail (Thermo Scientific), 50 mmol L^−1^ NaF and PMSF. The cell and tissue lysates were incubated at 4 °C for 30 min with rocking, and samples were sonicated and span at 12 000 rpm for 10 min. An equal amount of supernatant protein was loaded to SDS‐PAGE gel and transferred to a nitrocellulose (NC) membrane. The membrane was blocked with 5% milk/TBST (50 mm Tris‐HCl, pH 7.4 with 20 mm Tween 20) for 1 h at room temperature. Primary antibodies were diluted in 3% BSA/TBST and incubated with the NC membrane overnight at 4 °C. The blotted membrane was washed in TBST three times (each time 10 min), followed by incubation with HRP‐conjugated secondary antibodies in 5% milk/TBST for 1 h at room temperature. Western blot images were developed with ECL and acquired with a ChemiScop 6000 (CLiNqinxiang, Shanghai, China). Images were quantified with densitometric quantitation and analyzed using ImageJ software.

### Lysosome Activity Assay

The lysosomal activity was assayed with a commercial enzyme‐linked immunosorbent assay kit (https://shjingk.biomart.cn/, Shanghai, China, JLC21595 and JLC1595) by following the manufacturer's directions. Cells and mouse brain tissues were homogenized in NP40 buffer (50 mm Tris, pH7.4, 150 mm NaCl, 1% NP‐40, and protease and phosphatase inhibitors) at 0.5 µg mL^−1^, and 50 µL of the protein samples were added in triplicate into each well coated with the primary mouse anti‐lysosome enzyme antibody. The plate was incubated at 37 °C for 30 min and was washed 5 times with washing buffer. Ready‐to‐use HRP‐labeled secondary anti‐Lysosome enzyme antibody (50 µL) was added into each well, and the plate was incubated at 37°C for 30 min. The plate was washed five times, and 50 µL of the developing solution A containing the 3,3′,5,5′‐Tetramethylbenzidine (TMB) was added into each well for incubation at 37 °C for 10 min, followed by adding 50 µL of the stopping solution. The plate was read at OD_450nm_ in the H1 Biotek plate reader, and the lysosome activity was calculated based on the standard curve that was generated according to the manufacture instruction.

### Human Huntingtin ELISA Analysis

For the quantitative detection of human HTT, we utilized a human Huntington's enzyme‐linked immunosorbent assay (ELISA) kit (CSB‐EL010905HU) from Cusabio (Wuhan, China). The detection of human HTT in cells and mouse tissues was performed following the manufacturer's instructions. Cells and mouse brain tissues were homogenized in NP40 buffer (50 mm Tris, pH 7.4, 150 mm NaCl, 1% NP‐40, and protease and phosphatase inhibitors) at a concentration of 0.5 mg mL^−1^. Then, 100 µL of standards and samples were added to each well of the ELISA plate. The plate was incubated for 2 h at 37 °C. After removing the liquid from each well, 100 µL of Biotin‐antibody was added to each well, and the plate was incubated for 1 h at 37 °C. The plate was washed three times, and then 100 µL of HRP‐avidin was added to each well. The plate was incubated for 1 h at 37 °C and washed five times with washing buffer. Next, 90 µL of TMB Substrate was added to each well and incubated for 15–30 min at 37 °C, protected from light. Finally, 50 µL of Stop Solution was added to each well, and the plate was gently tapped to ensure thorough mixing. The plate was read at OD450nm using the H1 Biotek plate reader. The quantification of human huntingtin was calculated based on the standard curve generated according to the manufacturer's instructions.

### Cell Viability Analysis

For the detection of cell viability, we utilized the Cell Counting Kit‐8 (CCK‐8) assay (GK10001) from GLP Bio (https://www.glpbio.cn/). The assay was performed following the manufacturer's instructions. Transfected cells were cultured in 96‐well plates with 100 µL of culture medium and incubated at 37 °C in a CO_2_ incubator for 24 h. Subsequently, 10 µL of CCK‐8 solution was added to each well using a multichannel pipette, and the plates were further incubated for 1–4 h in the incubator. The absorbance of the plate was measured at OD450nm using the H1 Biotek plate reader. Cell viability (%) was calculated using the following formula: Cell viability (%) = [(As‐Ab) / (Ac‐Ab)] × 100, where As represents the absorbance of the experimental wells containing cells, culture medium, CCK‐8, and the tested compound; Ab represents the absorbance of the blank wells containing culture medium and CCK‐8; and Ac represents the absorbance of the control wells containing cells, culture medium, and CCK‐8.

### Immunofluorescent Staining

Mice were anesthetized with isoflurane 1 ml L^−1^ space and transcardially perfused with 0.9% saline, then brain were dissected. The dissected half brain was fixed with 10 mL of 4% paraformaldehyde for 24 h, and then dehydrate with 30% sucrose for 48 h at 4 °C. After embedding with tissue cryoprotectant (OCT), brains were sectioned into 20 µm slices using a cryostat (Leica CM1950).

For immunofluorescent staining, brain slices mounted on a pre‐coated glass slide were prefixed with 4% paraformaldehyde for 10 min, then blocked with 2% goat serum and 0.1% TrionX‐100 in 3% BSA for 1 h at room temperature. The samples were then incubated with primary antibodies in the blocking buffer in a staining tray (Thermo‐fisher) overnight at 4 °C. After removing primary antibodies, samples were washed three times with PBS and were incubated with respective fluorescent labeled secondary antibodies (anti‐rabbit, anti‐mouse, or anti‐rat Alexa Fluor 488, 555, or 647; Themo‐Fisher Scientific) for 60 min at room temperature. Imaging acquisition was done by using a confocal imaging system (Olympus FV3000 Microscope, Japan) or TissueFAXS PLUS (TissueGnostics, Vienna, AUT).

Cultured cells were fixed with 4% paraformaldehyde for 10 min, and then blocked with 2% goat serum and 0.1% TrionX‐100 in 3% BSA for 1 h at room temperature, and then incubated with primary antibodies overnight at 4 °C. After removing primary antibodies, cells were washed three times with PBS and incubated with secondary antibodies for 60 min at room temperature. Fluorescent images were obtained using a Zeiss microscope (Axiovert 200 MOT) with a digital camera (Hamamatsu Orca‐100) and Openlab software (Improvision Inc.) or a confocal imaging system (Olympus FV3000 Microscope) and a TissueFAXS PLUS (TissueGnostics, Vienna, AUT).

### Mice

All animal procedures were approved by the Institutional Animal Care and Use Committee of Jinan University (Approval No.:IACUC‐20221117‐03). HTT knock‐in (140Q) mice expressing full length mHTT containing human exon 1 with 140 CAGs were from JacksonLab (^#^027409). Mice were housed in the Division of Animal Resources at Jinan University on a 12‐h light/dark cycle. Heterozygous 140Q KI mice were then produced by mating male heterozygous mice with female wild‐type C57BL/6J mice. All procedures and husbandry were in accordance with the NIH Guideline for the Care and Use of Laboratory Animals.

### Brain Stereotaxic and Intravenous Injection of AAV

For AAV used in brain stereotaxic and intravenous injections, AAV‐PHP. eB: SM3 and control virus AAV‐PHP. eB: GFP were packaged and purified by PackGene Biotech (PackGene, Guangzhou, China). For brain stereotaxic injection, 6‐month‐old HTT 140Q KI mice and controls were anesthetized with isoflurane inhalation, and 1 × 10^10^ genome copies (GC) virus per mouse (1 µL of 10^13^ GC mL^−1^) were injected. Stereotaxic injection of AAV into mouse brains was performed using the method described in our previous studies.^[^ ^27^ ^]^ In brief, the head of mouse was placed in a Kopf stereotaxic frame (Model 1900) equipped with a digital manipulator, a UMP3–1 Ultra pump, and a 10 µL Hamilton microsyringe (Hamilton Co., Reno, NV, USA), and a 33G needle was inserted through a 1 mm drilled hole through the scalp. The following coordinates (relative to the bregma) were used to target the striatum region of the brain: anteroposterior (AP), +0.55 mm; mediolateral (ML), ±2 mm; dorsoventral (DV), −3.5 mm. The mice were euthanized 1 month after virus injection.

For intravenous delivery, HD KI‐140Q and control mice at 6 months of age were anaesthetized and injected with virus retro‐orbitally at a dose of 5×10^11^ genome copies (GC) virus per mouse (50 µL of 10^13^ GC mL^−1^). Mice were euthanized at 7 months of age to analyze HD pathologies. All surgical procedures were performed in a designated procedure room and in accordance with the Guidelines for the Care and Use of Laboratory Animals and biosafety procedures at Jinan University.

### Cell Culture

Human embryonic kidney (HEK) 293T (293T) cells and HTT‐120Q stably transfected HEK293 cell line were maintained in growth medium consisting of Dulbecco's Modified Eagle Medium (DMEM) supplemented with 10% fetal bovine serum and 1% penicillin/ streptomycin solution. Cells were incubated at 37 °C in 5% CO_2_. For in vitro testing of the ability of SM3 to clear mutant huntingtin, 293T cells were divided into three groups, control, mHTT only and mHTT + SM3 groups. The control group was transfected with plasmid expressing the HA epitope only, the mHTT group was co‐transfected with HTT N171‐150Q, and the mHTT+SM3 group was co‐transfected with HTT N‐171‐150Q and SM3. The HTT‐120Q stably transfected HEK293 cell lines were also divided into these three groups transfected with the same plasmids. Transfection concentrations are N171‐150Q plasmid 5 µg per dish, SM3 plasmid 12 µg per dish in 10 cm cell culture dish. At 48 h after transfection, cells were harvested for western blotting and immunofluorescence analysis.

To determine the mHTT degradation pathway, we used the proteasome inhibitor MG132 and the lysosome inhibitor bafilomycin A1 (bafA1). The mHTT+SM3 group was divided into three groups. After transfection for 5 h, the culture medium was replaced with medium containing 1/1000 DMSO (control group), 10 µm MG132, or 200 nm bafA1. The culture dishes were then incubated at 37 °C in a CO_2_ incubator for 48 h, cells were harvested for western blotting and immunofluorescence analysis.

### Mouse Behavioral Tests

All animal tests were performed in accordance with the Guidelines for the Care and Use of Laboratory Animals and biosafety procedures at Jinan University. Animal behavior tests were performed using at least 12 mice per group and both male and female mice were included. Mouse rotarod behavior was assessed using a rotarod apparatus (Rotamex 4/8, Columbus Instruments International). Mice were trained on the rotarod at 20 RPM for 5 min for three consecutive days. For testing, the rotarod was gradually accelerated to 40 RPM over a 5‐min period. Latency to fall was recorded for each trial. Each mouse went through three trials, and the average data of each group was analyzed (*n* = 12 mice per group). The balance beam apparatus consists of 1 m beams with a flat surface of 6 mm width resting 50 cm above the top of the bench on two poles. A black box at the end of the beam is the finish point. The total running distance was roughly 0.8 m. Prior to data collection, each mouse was trained for 3 consecutive days with 3 runs per day.^[5g]^ For the grip strength test, Mice were allowed to grip the metal grids of a grip meter (Ametek Chatillon) with all their limbs, and they were gently pulled backwards by the tail until they could no longer hold the grids. The peak grip strength observed in 5 trials was recorded and averaged.^[^ ^28^ ^]^ For Catwalk, we used a small animal gait analyzer (CatWalk XT, Noldus, Netherlands) for detection, and the mice were trained for 3 days before the experiment. The time required for the mouse to freely pass through the set length of the detection channel during detection, and its average speed were calculated to evaluate the movement of the mouse under the condition of natural walking. This process was all completed in a dark room environment, and each mouse was tested at least three times.

### RNA‐Seq and Data Analysis

The total RNA of the striatum in GFP‐injected WT or HD KI‐140Q mice or SM3‐injected HD KI‐140Q mice was isolated using RNAiso Plus (TaKaRa, Japan). The RNAs were sent to HeQin Biotechnology Corporation in Guangzhou, China, for RNA‐seq analysis and database construction. A total of 2 µg of RNA per sample was used for analysis. NEBNext Ultra RNA Library Prep Kit for Illumina (E7530L; NEB) was used for sequencing following the manufacturer's recommendations. After cluster generation, the libraries were sequenced and 150‐bp paired‐end reads were generated using Illumina platform. After obtaining the raw sequencing data, Trimmomatic software was used to control the quality of raw RNA‐seq data and trim the sequencing adapter.^[^ ^29^ ^]^ Then STAR software was used to map the clean data to the mouse genome, which was downloaded from the Ensembl website, to obtain the sam files.^[^ ^30^ ^]^ The samtools was used to convert sam files into bam files, sort and build index files.^[^ ^31^ ^]^ Stringtie and its script “prepDE.py” was used to quantify genes and convert them into read counts matrix.^[^ ^32^ ^]^ Finally, the R package DESeq2 was used for gene differential expression analysis, and the read counts matrix was used as the input file.^[^ ^33^ ^]^ Genes with adjusted P‐value < 0.01 and an absolute fold change > 2 were considered as DEGs. GO enrichment analysis for DEGs in a group was carried out using TBtools.^[^ ^34^ ^]^ GO terms with a *P* value < 0.01 and a hit rate > 0.05 were considered significantly enriched.

### Statistical Analysis

When two groups were compared, a two‐tailed Student's t‐test was used to assess the statistical significance. One‐way ANOVA with Dunnett's multiple comparisons test was used to assess the statistical significance when analyzing multiple groups. Data are presented as mean ± SEM. For biochemical and histological studies, we used at least three mice per group. For animal behavioral studies, 12 mice per genotype were used. More than three independent experiments were done to obtain the blots or micrographic results that were used for figure presentations, and the representative results were shown in figures. The quantification of Western blots was performed by Image J. Calculations were performed with GraphPad Prism8 software. A p‐value < 0.05 was considered significant.

### Study Approval

All experimental procedures on mice were approved by the Institutional Animal Care and Use Committee of Jinan University (Approval No.:IACUC‐20221117‐03).

## Conflict of interest

The authors declare no conflict of interest.

## Author Contributions

C.L. and Y.L. contributed equally to this work. S.Y., X.J.L., and S.H.L. designed the research. C.J.L., Y.Q.L., Y.Z.C., X.C.S., X.Z., J.W.L., J.H., X.S.C., C.H.H., W.W., J.H.W., J.X.W., J.L.G., and Z.C.T. performed the experiments. S.Y., X.J.L., S.H.L., and C.J.L. analyzed the data. Y.Z.C. and J.W.L. performed bioinformatics analysis. C.J.L., S.Y., X.J.L., and S.H.L. wrote the paper with input from all authors.

## Supporting information

Supporting Information

## Data Availability

The data that support the findings of this study are available from the corresponding author upon reasonable request.

## References

[1] a) F. O. Walker , Semin Neurol 2007, 27, 143; 17390259 10.1055/s-2007-971176

[2] a) M. E. MacDonald , C. M. Ambrose , M. P. Duyao , R. H. Myers , C. Lin , L. Srinidhi , G. Barnes , S. A. Taylor , M. James , N. Groot , H. MacFarlane , B. Jenkins , M. A. Anderson , N. S. Wexler , J. F. Gusella , G. P. Bates , S. Baxendale , H. Hummerich , S. Kirby , M. North , S. Youngman , R. M. Gunther Zehetner , Z. Sedlacek , A. Poustka , A.‐M. Frischauf , H. Lehrach , A. J. Buckler , D. Church , L. Doucette‐Stamm , M. C. O'Donovan , et al., Cell 1993, 72, 971;8458085

[3] a) J. Bradford , J. Y. Shin , M. Roberts , C. E. Wang , X. J. Li , S. Li , Proc Natl Acad Sci 2009, 106, 22480; 20018729 10.1073/pnas.0911503106 PMC2799722

[4] J. P. Vonsattel , R. H. Myers , T. J. Stevens , R. J. Ferrante , E. D. Bird , E. P. Richardson, Jr. , J Neuropathol Exp Neurol 1985, 44, 559. 2932539 10.1097/00005072-198511000-00003

[5] a) Z. J. Chen , B. T. Kren , P. Y. Wong , W. C. Low , C. J. Steer , Biochem. Biophys. Res. Commun. 2005, 329, 646; 15737634 10.1016/j.bbrc.2005.02.024

[6] P. O. Bauer , A. Goswami , H. K. Wong , M. Okuno , M. Kurosawa , M. Yamada , H. Miyazaki , G. Matsumoto , Y. Kino , Y. Nagai , N. Nukina , Nat. Biotechnol. 2010, 28, 256. 20190739 10.1038/nbt.1608

[7] R. Y. He , X. M. Lai , C. S. Sun , T. S. Kung , J. Y. Hong , Y. S. Jheng , W. N. Liao , J. K. Chen , Y. F. Liao , P. H. Tu , J. J. Huang , Adv. Sci. (Weinh) 2020, 7, 1901165. 31993280 10.1002/advs.201901165 PMC6974936

[8] a) N. R. Franich , H. L. Fitzsimons , D. M. Fong , M. Klugmann , M. J. During , D. Young , Mol. Ther. 2008, 16, 947; 18388917 10.1038/mt.2008.50 PMC3793641

[9] K. Kingwell , Nat Rev Drug Discov 2021, 20, 412. 34012000 10.1038/d41573-021-00088-6

[10] a) A. S. Lo , Q. Zhu , W. A. Marasco , Handbook of Experimental Pharmacology, Vol. 181, Springer, Berlin, Heidelberg 2008, p. 343; 10.1007/978-3-540-73259-4_15 18071953

[11] a) C. Zhou , S. Przedborski , Biochim. Biophys. Acta 2009, 1792, 634; 18834937 10.1016/j.bbadis.2008.09.001 PMC2745095

[12] a) A. Messer , D. C. Butler , Neurobiol Dis 2020, 134, 104619; 31669671 10.1016/j.nbd.2019.104619

[13] a) A. L. Southwell , J. Ko , P. H. Patterson , J. Neurosci. 2009, 29, 13589; 19864571 10.1523/JNEUROSCI.4286-09.2009 PMC2822643

[14] a) D. C. Butler , A. Messer , PLoS One 2011, 6, e29199; 22216210 10.1371/journal.pone.0029199 PMC3245261

[15] K. F. Lu , F. den Brave , S. Jentsch , Autophagy 2017, 13, 1799. 28813181 10.1080/15548627.2017.1358851 PMC5965392

[16] F. G. Guarnieri , L. M. Arterburn , M. B. Penno , Y. Cha , J. T. August , J. Biol. Chem. 1993, 268, 1941. 8420968

[17] R. R. Paudel , D. Lu , S. Roy Chowdhury , E. Y. Monroy , J. Wang , Biochemistry 2023, 62, 564. 36130224 10.1021/acs.biochem.2c00310 PMC10245383

[18] a) L. B. Menalled , J. D. Sison , I. Dragatsis , S. Zeitlin , M. F. Chesselet , J Comp Neurol 2003, 465, 11; 12926013 10.1002/cne.10776

[19] a) C. H. Lin , S. Tallaksen‐Greene , W. M. Chien , J. A. Cearley , W. S. Jackson , A. B. Crouse , S. Ren , X. J. Li , R. L. Albin , P. J. Detloff , Hum. Mol. Genet. 2001, 10, 137; 11152661 10.1093/hmg/10.2.137

[20] M. A. Hickey , A. Kosmalska , J. Enayati , R. Cohen , S. Zeitlin , M. S. Levine , M. F. Chesselet , Neuroscience 2008, 157, 280. 18805465 10.1016/j.neuroscience.2008.08.041 PMC2665298

[21] a) K. Y. Chan , M. J. Jang , B. B. Yoo , A. Greenbaum , N. Ravi , W. L. Wu , L. Sanchez‐Guardado , C. Lois , S. K. Mazmanian , B. E. Deverman , V. Gradinaru , Nat. Neurosci. 2017, 20, 1172; 28671695 10.1038/nn.4593 PMC5529245

[22] a) S. Malaiya , M. Cortes‐Gutierrez , B. R. Herb , S. R. Coffey , S. R. W. Legg , J. P. Cantle , C. Colantuoni , J. B. Carroll , S. A. Ament , J. Neurosci. 2021, 41, 5534; 34011527 10.1523/JNEUROSCI.2074-20.2021 PMC8221598

[23] a) F. Saudou , S. Humbert , Neuron 2016, 89, 910; 26938440 10.1016/j.neuron.2016.02.003

[24] G. Gallardo , C. H. Wong , S. M. Ricardez , C. N. Mann , K. H. Lin , C. E. G. Leyns , H. Jiang , D. M. Holtzman , Mol Neurodegener 2019, 14, 38. 31640765 10.1186/s13024-019-0340-6 PMC6805661

[25] Z. Li , C. Wang , Z. Wang , C. Zhu , J. Li , T. Sha , L. Ma , C. Gao , Y. Yang , Y. Sun , J. Wang , X. Sun , C. Lu , M. Difiglia , Y. Mei , C. Ding , S. Luo , Y. Dang , Y. Ding , Y. Fei , B. Lu , Nature 2019, 575, 203. 31666698 10.1038/s41586-019-1722-1

[26] S. M. Banik , K. Pedram , S. Wisnovsky , G. Ahn , N. M. Riley , C. R. Bertozzi , Nature 2020, 584, 291. 32728216 10.1038/s41586-020-2545-9 PMC7727926

[27] C. Huang , J. Li , G. Zhang , Y. Lin , C. Li , X. Zheng , X. Song , B. Han , B. Guo , Z. Tu , J. Zhang , Y. Sun , Y. Wang , Z. Zhang , S. Yan , Hum. Mol. Genet. 2021, 30, 1484. 33929499 10.1093/hmg/ddab101

[28] H. Yang , S. Yang , L. Jing , L. Huang , L. Chen , X. Zhao , W. Yang , Y. Pan , P. Yin , Z. S. Qin , B. Tang , S. Li , X. J. Li , Nat. Commun. 2020, 11, 2582. 32444599 10.1038/s41467-020-16318-1 PMC7244548

[29] A. M. Bolger , M. Lohse , B. Usadel , Bioinformatics 2014, 30, 2114. 24695404 10.1093/bioinformatics/btu170 PMC4103590

[30] a) A. Dobin , C. A. Davis , F. Schlesinger , J. Drenkow , C. Zaleski , S. Jha , P. Batut , M. Chaisson , T. R. Gingeras , Bioinformatics 2013, 29, 15; 23104886 10.1093/bioinformatics/bts635 PMC3530905

[31] H. Li , B. Handsaker , A. Wysoker , T. Fennell , J. Ruan , N. Homer , G. Marth , G. Abecasis , R. Durbin , Bioinformatics 2009, 25, 2078. 19505943 10.1093/bioinformatics/btp352 PMC2723002

[32] M. Pertea , G. M. Pertea , C. M. Antonescu , T. C. Chang , J. T. Mendell , S. L. Salzberg , Nat. Biotechnol. 2015, 33, 290. 25690850 10.1038/nbt.3122 PMC4643835

[33] M. I. Love , W. Huber , S. Anders , Genome Biol. 2014, 15, 550. 25516281 10.1186/s13059-014-0550-8 PMC4302049

[34] C. Chen , H. Chen , Y. Zhang , H. R. Thomas , M. H. Frank , Y. He , R. Xia , Mol. Plant 2020, 13, 1194. 32585190 10.1016/j.molp.2020.06.009

