# Supplementary material for: A Specific Mini‐Intrabody Mediates Lysosome Degradation of Mutant Huntingtin
Source: Adv Sci (Weinh). 2023 Sep 8;10(31):2301120. doi: 10.1002/advs.202301120 (PMC10625127; doi:10.1002/advs.202301120)
Supplement: Supplementary file 1 — Supporting Information [file ADVS-10-2301120-s001.pdf]

ADVANCED SCIENCE
Open Access

# Supporting Information

for *Adv. Sci.*, DOI 10.1002/advs.202301120

A Specific Mini-Intrabody Mediates Lysosome Degradation of Mutant Huntingtin

*Caijuan Li, Yingqi Lin, Yizhi Chen, Xichen Song, Xiao Zheng, Jiawei Li, Jun He, Xiusheng Chen, Chunhui Huang, Wei Wang, Jianhao Wu, Jiaxi Wu, Jiale Gao, Zhuchi Tu, Xiao-Jiang Li*, Sen Yan* and Shihua Li**

**Supplementary Materials**

# A specific mini-intrabody mediates lysosomal degradation of mutant huntingtin

**Authors:** Caijuan Li[1,2†], Yingqi Lin[1,2†], Yizhi Chen[1], Xichen Song[1], Xiao Zheng[1], Jiawei Li[1], Jun He[3], Xiusheng Chen[1], Chunhui Huang[1], Wei Wang[1], Jianhao Wu[1], Jiaxi Wu[1], Jiale Gao[1], Zhuchi Tu[1], Xiao-Jiang Li[1*], Sen Yan[1,2*], Shihua Li[1*]

**Affiliations:**

[1]Guangdong Key Laboratory of Non-human Primate Research, Key Laboratory of CNS Regeneration (Ministry of Education), GHM Institute of CNS Regeneration, Jinan University, Guangzhou, China 510632.

[2]School of Medicine, Jinan University, Guangzhou 510632, China.

[3]Institute of Laboratory Animal Science, Jinan University, Guangzhou, China.

*Corresponding authors:

xjli33@jnu.edu.cn;231yansen@163.com;lishihualis@jnu.edu.cn

[†]These authors contributed equally to this work

**Content**

**Supplementary Figures 1-7**

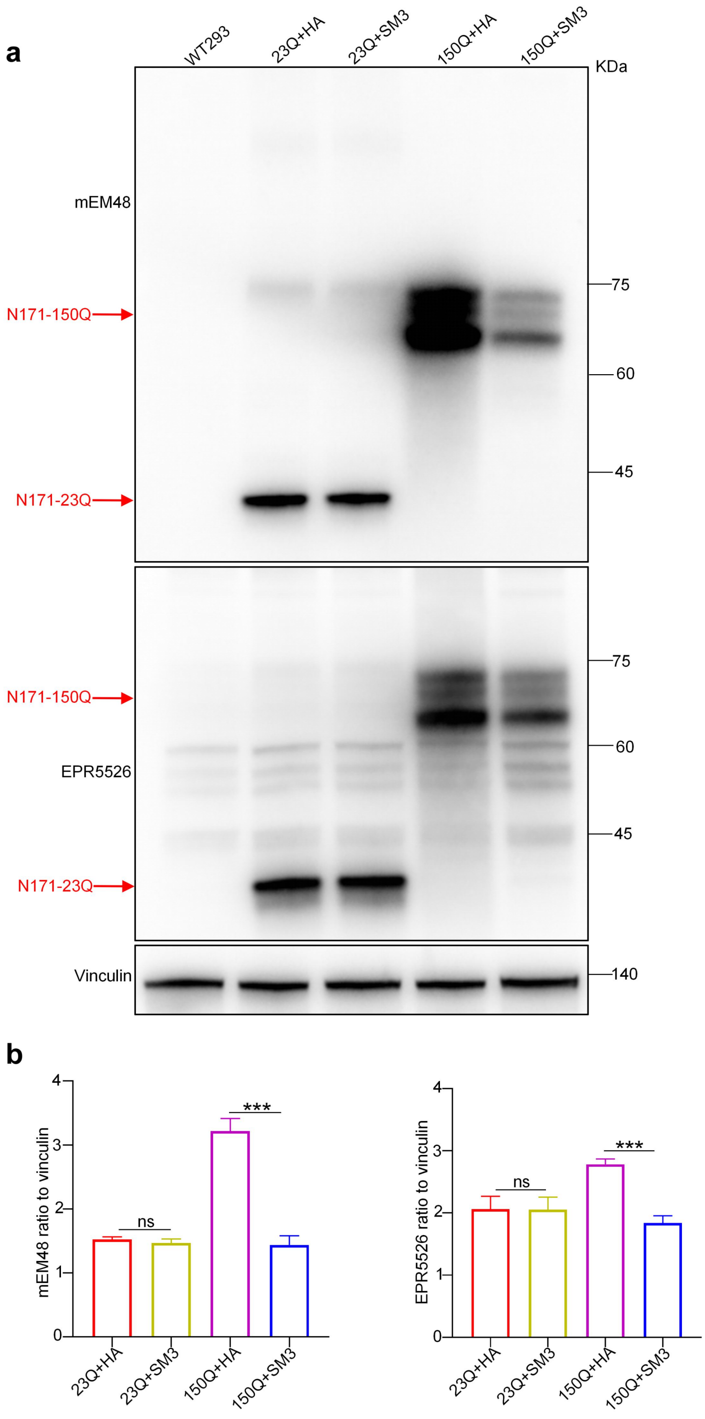


**Figure S1. Comparison of N-terminal HTT-23Q and -150Q binding to SM3. a)** Western blotting analysis of the transfected 293T cells and HTT N171-23Q or -150Q with two anti-HTT antibodies. **b)** Quantification of the ratios of HTT recognized by HTT antibodies (mEM48 and EPR5526) to vinculin on the western blots. The data were obtained from four independent experiments (n = 4). Data were analyzed by Student's t test and presented as mean ± SEM. ****P*=0.0003 (mEM48); ****P*=0.0006 (EPR5526).

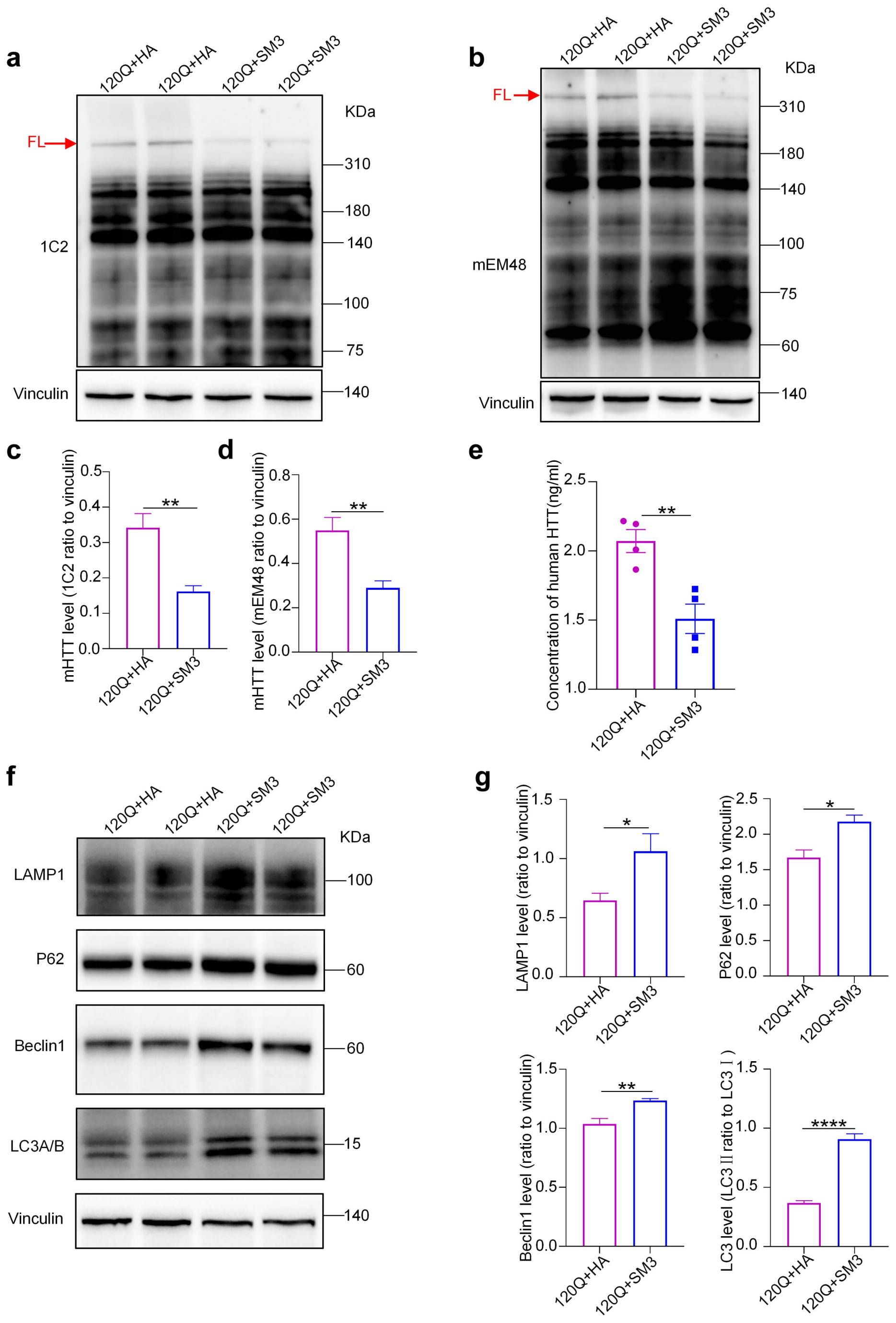


**Figure S2. Analysis of the effect of SM3 on the full-length HTT-120Q transfected 293 cells. a, b)** Western blotting analysis of the full-length HTT-120Q stably expressed 293T cells, which were transfected with SM3 or vector expressing HA only. Full-length mHTT (arrow pointed) were detected with 1C2 or mEM48 antibodies. **c, d)** Quantitation of the ratios of mHTT detected by 1C2 or mEM48 to vinculin on the western blots. The data were obtained from four independent experiments (n = 4). Data are analyzed by two-tailed Student's t test and presented as mean ± SEM; ***P* = 0.0058 (1C2); ***P* = 0.0081(mEM48). **e)** Quantification of human HTT using ELISA. n = 4 per group. Data were analyzed by two-tailed Student's t test and presented as mean ± SEM. **P=0.0058. **f)** Western blot analysis of HTT-120Q 293 cells transfected with SM3 or the vector expressing HA only. The

blots were probed with LAMP1, P62, Beclin 1 and LC3 antibodies. Vinculin served as a loading control. **g)** Quantitation of the ratios of LAMP1, P62 and Beclin 1 to vinculin on the western blots, and quantitation of the ratios of LC3II to LC3I (LC3A/B) on the western blots. The data were obtained from four independent experiments (n = 4). Data are analyzed by two-tailed Student's t test and presented as mean ± SEM. LAMP1: **P* = 0.0304. P62: **P* = 0.0124. Beclin 1: ***P* = 0.007. LC3: *****P*<0.0001.

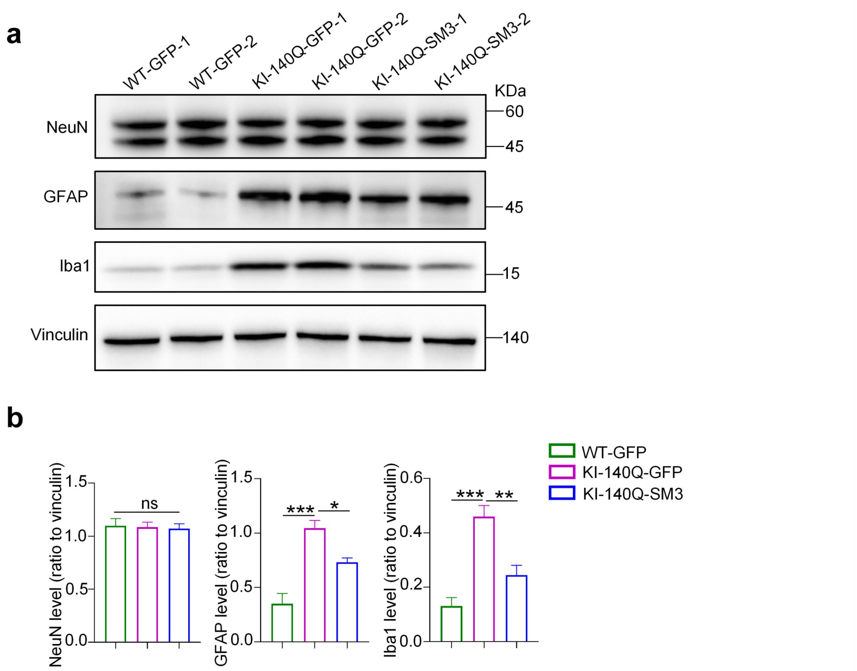


**Figure S3. Analysis of the HD KI-140Q mouse brain injected with SM3. a)** Western blotting analysis of the expression of neuronal (NeuN) and glial (GFAP, Iba1) proteins in the striatum of HD KI-140Q and wild type (WT) mice injected with GFP (control) or SM3 at 7 months of age. **b)** Quantification of the ratios of NeuN, GFAP, and Iba1 to vinculin on the western blots. The data were obtained from four independent experiments (n = 4). Data were analyzed by one-way ANOVA with Dunnett's multiple comparisons test and are presented as mean ± SEM. GFAP: **P* =0.0249; ****P* = 0.0002. Iba1: ***P* = 0.0044; ****P* = 0.0003.

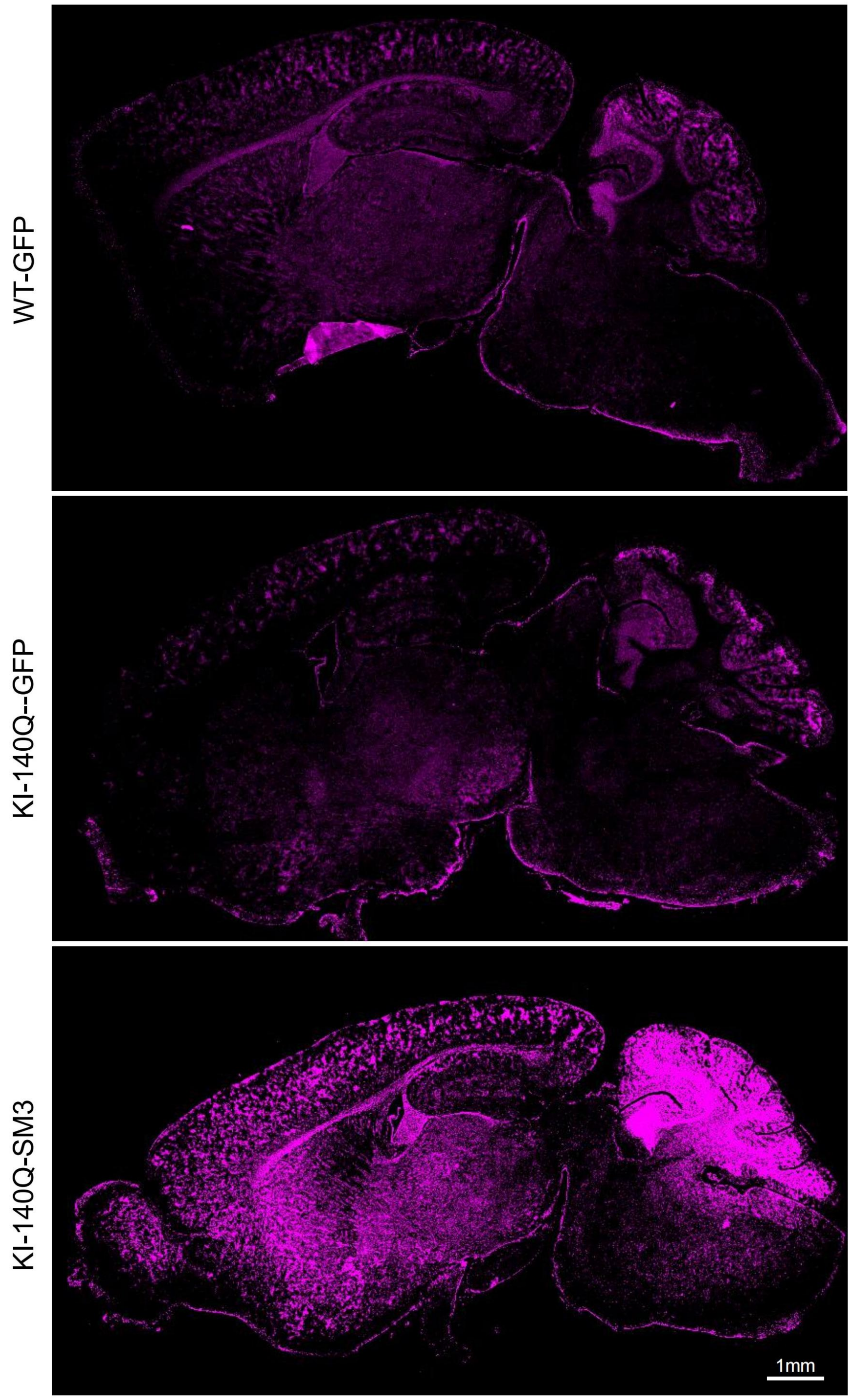


**Figure S4. Sagittal images of HA antibody immunofluorescent staining of SM3 in the HD KI-140Q mouse brain.**

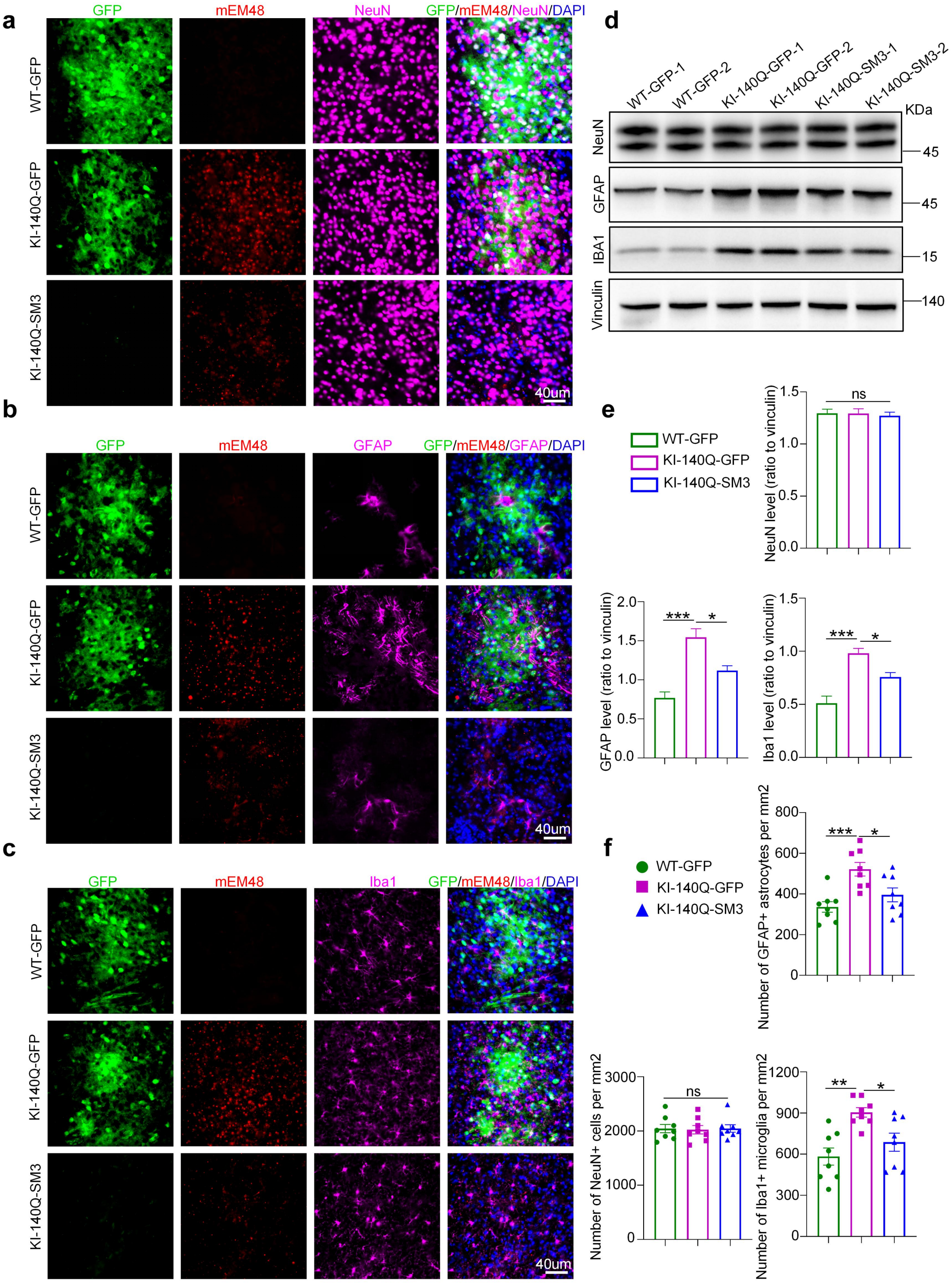


**Figure S5. Analysis of HD KI-140Q mouse brain after intravenous injection of SM3.** Immunostaining and western blotting of HD KI-140Q mouse striatum one month after intravenous injection of SM3. **a-c)** Representative immunofluorescent fluorescent images of the striatum from GFP or SM3 injected HD KI-140Q mice. Antibodies for NeuN, GFAP and Iba1 were used. All the sections were stained with mEM48 and anti-GFP as well as DAPI. GFP injection was used as control. Note that the numbers of NeuN-positive neurons did not change in HD KI-140Q mice in all treatments. SM3 injection significantly reduced gliosis

shown by GFAP or Iba1staining. Scale bars: 40 μm. **d)** Western blotting analysis of neuronal (NeuN) and glial (GFAP, Iba1) proteins in the HD KI-140Q or WT mice injected with GFP or SM3. **e)** Quantification of the ratios of NeuN, GFAP, and Iba1 to vinculin on the western blots. The data were obtained from four independent experiments (n = 4). Data were analyzed by one-way ANOVA with Dunnett's multiple comparisons test and presented as mean ± SEM. GFAP: ***P= 0.0002; *P= 0.0107. Iba1: ***P= 0.0003; *P= 0.0257. **f)** Quantification of the NeuN, GFAP, Iba1 immunostaining. Data are analyzed by one-way ANOVA with Dunnett's multiple comparisons test and presented as mean ± SEM. *n*= 8 mice per group; GFAP: **P* = 0.0183 (WT-GFP verses HD KI-140Q+SM3); ****P* = 0.0008 (WT-GFP verses HD KI-140Q). Iba1: **P* = 0.0228 (WT-GFP verses HD KI-140Q+SM3); ***P* = 0.0011 (WT-GFP verses HD KI-140Q).

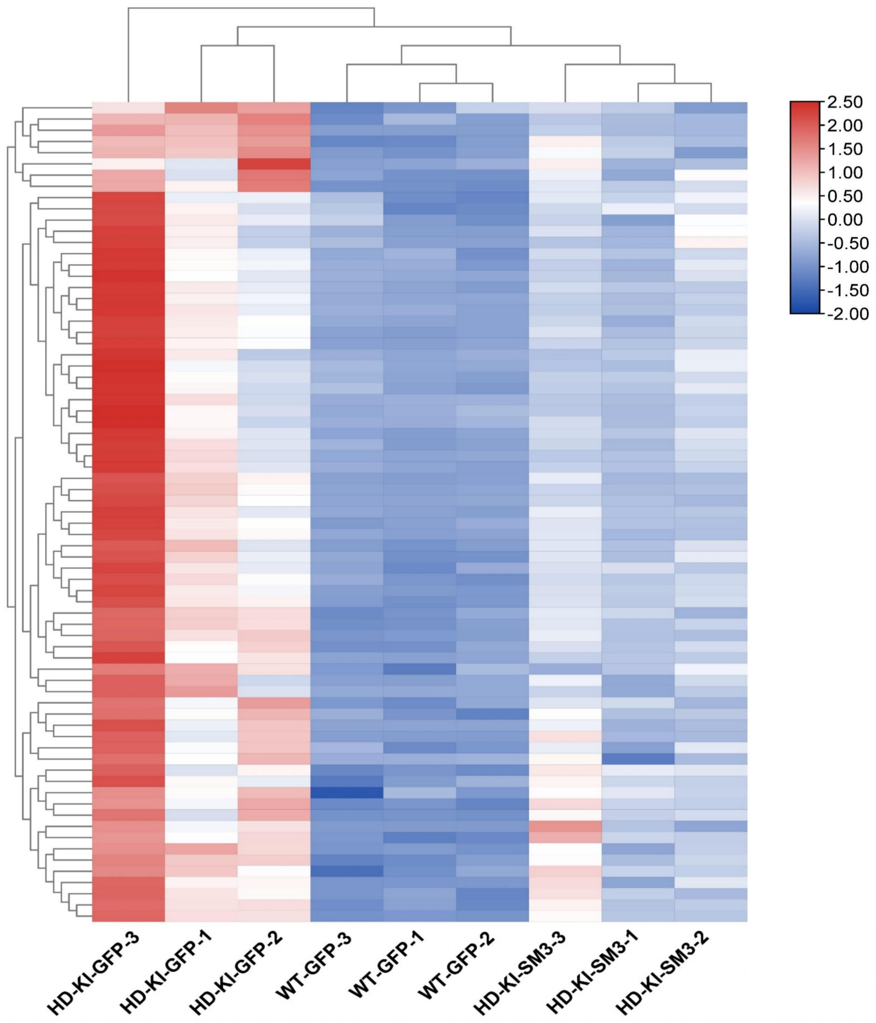


**Figure S6. RNA-seq analysis of HD-KI mice after SM3 treatment.** Total RNAs from WT and HD KI-140Q treated with GFP or SM3 were examined. The striatum from three animals of each group were examined. The mice at 6 months of age were stereotaxically injected with AAV and then their striatal tissues were isolated for RNA-seq analysis one month after injection. The heat map of the RNA-seq analysis reveals the alterations in the transcripts related to immune and inflammatory responses in HD KI mouse brain.

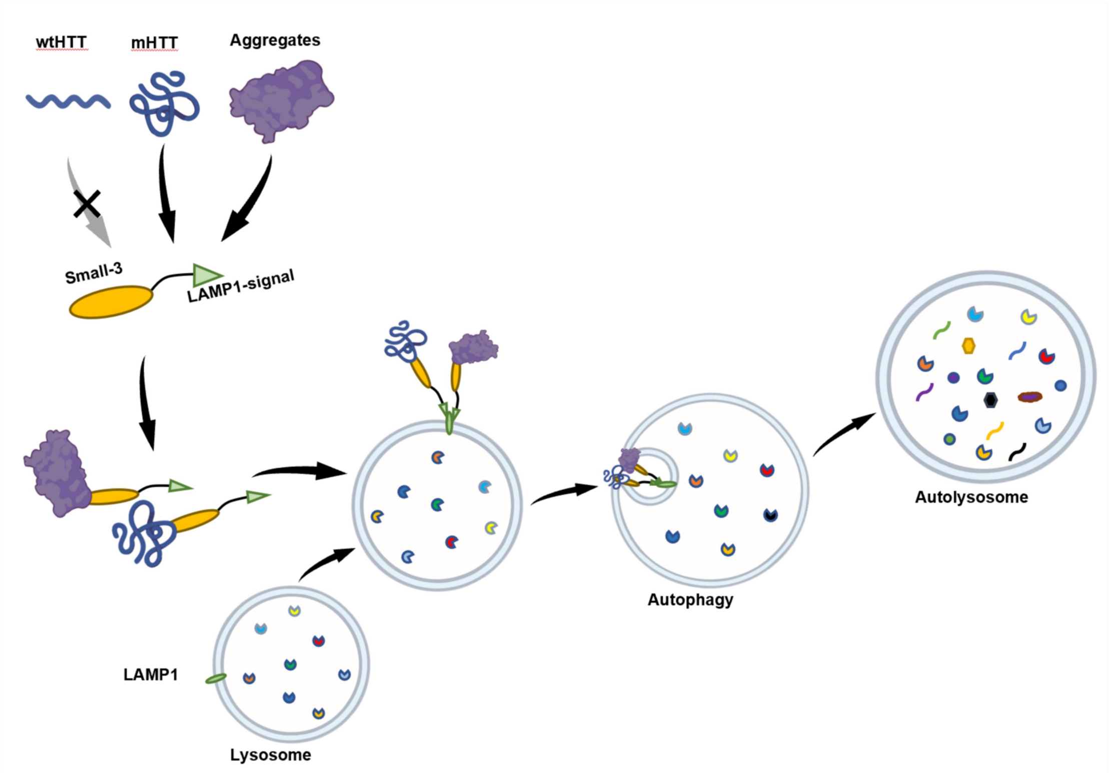


**Figure S7. A proposed model of how SM3 induces mHTT degradation.** A small peptide derived from HTT intrabody and linked with the lysosomal targeting signal can selectively bind mHTT and bring it to the lysosome for degradation.
